# Hospital spending and length of stay attributable to perioperative adverse events for inpatient hip, knee, and spine surgery: a retrospective cohort study

**DOI:** 10.1186/s12913-023-10055-z

**Published:** 2023-10-25

**Authors:** Y. Raja Rampersaud, Kala Sundararajan, Shgufta Docter, Anthony V. Perruccio, Rajiv Gandhi, Diana Adams, Natasha Briggs, J. Rod Davey, Michael Fehlings, Stephen J. Lewis, Rosalie Magtoto, Eric Massicotte, Angela Sarro, Khalid Syed, Nizar N. Mahomed, Christian Veillette

**Affiliations:** 1 Division of Orthopaedic Surgery, Schroeder Arthritis Institute, University Health Network, Toronto, ON Canada; 2 Krembil Research Institute, University Health Network, Toronto, ON Canada; 3 Department of Surgery, University of Toronto, Toronto, ON Canada; 4 Division of Neurosurgery, Krembil Neuroscience Centre, University Health Network, Toronto, ON Canada; 5 Institute of Health Policy, Management & Evaluation, Dalla Lana School of Public Health, University of Toronto, Toronto, ON Canada

**Keywords:** Orthopaedic joint surgery, Neurosurgery, Spine surgery, Inpatient admission, Health care costs, Length of stay, Complications, Adverse events, Economic evaluation

## Abstract

**Background:**

The incremental hospital cost and length of stay (LOS) associated with adverse events (AEs) has not been well characterized for planned and unplanned inpatient spine, hip, and knee surgeries.

**Methods:**

Retrospective cohort study of hip, knee, and spine surgeries at an academic hospital in 2011–2012. Adverse events were prospectively collected for 3,063 inpatient cases using the Orthopaedic Surgical AdVerse Event Severity (OrthoSAVES) reporting tool. Case costs were retrospectively obtained and inflated to equivalent 2021 CAD values. Propensity score methodology was used to assess the cost and LOS attributable to AEs, controlling for a variety of patient and procedure factors.

**Results:**

The sample was 55% female and average age was 64; 79% of admissions were planned. 30% of cases had one or more AEs (82% had low-severity AEs at worst). The incremental cost and LOS attributable to AEs were $8,500 (95% confidence interval [CI]: 5100–11,800) and 4.7 days (95% CI: 3.4–5.9) per admission. This corresponded to a cumulative $7.8 M (14% of total cohort cost) and 4,290 bed-days (19% of cohort bed-days) attributable to AEs. Incremental estimates varied substantially by (1) admission type (planned: $4,700/2.4 days; unplanned: $20,700/11.5 days), (2) AE severity (low: $4,000/3.1 days; high: $29,500/11.9 days), and (3) anatomical region (spine: $19,800/9 days; hip: $4,900/3.8 days; knee: $1,900/1.5 days). Despite only 21% of admissions being unplanned, adverse events in these admissions cumulatively accounted for 59% of costs and 62% of bed-days attributable to AEs.

**Conclusions:**

This study comprehensively demonstrates the considerable cost and LOS attributable to AEs in orthopaedic and spine admissions. In particular, the incremental cost and LOS attributable to AEs per admission were almost five times as high among unplanned admissions compared to planned admissions. Mitigation strategies focused on unplanned surgeries may result in significant quality improvement and cost savings in the healthcare system.

**Supplementary Information:**

The online version contains supplementary material available at 10.1186/s12913-023-10055-z.

## Background

Spine, hip, and knee surgeries are collectively the most common and resource-intensive surgical procedures performed in the United States and Canada [1–3]. In 2018, knee replacement, hip replacement, spinal fusion, femur fixation, and spinal discectomy were respectively the 2nd, 4th, 6th, 8th and 10th most frequent surgical procedures among inpatient hospital stays in the United States, and the three most costly operating room (OR) procedures in aggregate were spinal fusion, knee replacement, and hip replacement [1]. In Canada, hip replacement, knee replacement, fracture surgery, and disc surgery were respectively ranked the 2nd, 3rd, 4th, and 9th most common adult inpatient surgeries in 2020–2021 [2]. Hip and knee replacements alone account for $1.4 billion CAD annual spending, with volumes increasing by 5% annually before COVID-related disruptions in 2020 [3]. Similar trends have been noted in other OECD countries [4, 5]. Identifying specific macro- and micro-level cost drivers in orthopaedic and spine surgery is therefore a key step toward mitigating rising hospital costs—an issue of global significance.

Inpatient orthopaedic and spine procedures are often major surgical interventions with inherent risk of adverse events (AEs) [6] that can have substantial consequences for patients, providers and health systems [7–12]. Past literature examining AEs in orthopaedic surgery has relied on administrative data, which is known to underestimate AE incidence and lack information regarding their clinical context [13–18]. In North America, the American College of Surgeons National Surgical Quality Improvement Program (ACS-NSQIP) provides risk-adjusted surgical quality audit and feedback; it has been progressively implemented across the US and Canada. NSQIP has been shown to improve quality of care, reduce surgical morbidity and reduce AE-associated costs [19–21]. Despite increasing use of NSQIP and other large databases to study the orthopaedic population [22, 23], the relatively broad AE categories in these systems remain a concern regarding accurate identification of AEs specific to subspecialty procedures. Additionally, NSQIP does not classify AEs with respect to clinical severity, limiting more in-depth economic analysis. The validated Orthopaedic Surgical AdVerse Events Severity tool (OrthoSAVES) was developed to allow clinicians to accurately and reliably classify AEs relevant to the orthopaedic surgical population, and to grade AE severity using defined patient and process outcomes.

Our objective was to evaluate the incremental hospital expenditure and bed-days attributable to intra- and postoperative AEs in inpatient hip, knee, and spine surgery admissions, using the OrthoSAVES tool to capture and classify perioperative AEs. In particular, we aimed to quantify the cost and bed-days attributable to AEs in planned and unplanned admissions. We also stratified by AE severity grade and anatomical region. The findings from this study serve to further inform the economic rationale for implementing system-based surgical quality and reporting systems, and for subspecialty-focused strategies for AE identification and mitigation.

## Methods

### Study design

We undertook a retrospective cohort study with a cost analysis from the perspective of the hospital. The AE data collection method has been described by Millstone et al. [24] As part of a clinical quality initiative (QI), the OrthoSAVES tool (described below) was used to prospectively capture intra- and post-operative adverse events up to discharge for all orthopaedic and spine surgeries performed from January 2011 to December 2012 at a large academic hospital in Toronto, Canada. Patient and admission characteristics were obtained via retrospective chart review, including length of stay. Micro case costing data were obtained from the institution (described below in Cost data) and used to perform a health economic evaluation assessing the cost of AEs in orthopaedic surgery, overall and stratified by the factors noted above.

### Adverse event data (OrthoSAVES system)

A validated [25–27] AE reporting and severity grading system specific to orthopaedic and spine surgery is used for quality initiatives (QI) at our institution. Details of the Orthopaedic Surgical AdVerse Events System (OrthoSAVES) have been published [24] and its characteristics are summarized in Supplemental Table 1-A (see Additional File 1). OrthoSAVES relies on input from multiple members of the clinical team, rather than the attending surgeon alone. It defines an AE as any event that is due to medical or surgical management (not directly to the underlying disease process or injury) that results in harm to the patient or requires additional monitoring or treatment. The tool specifies six severity grades (Table 1), ranging from adverse events that require no treatment (grade 1) to adverse events resulting in death (grade 6). For this analysis, AEs grade 3 or higher were considered high-severity (i.e., likely to have a negative clinical impact on patient outcome).

**Table 1:** OrthoSAVES adverse event severity grades

Grade	Definition
1	Adverse event does not require treatment and has no adverse effect.
2	Adverse event requires simple or minor invasive treatment (e.g. Antibiotics, Foley catheter, nasogastric tube) and has no long-term effects on patient outcome.
3	Adverse event requires invasive (e.g. surgery) or complex treatment (e.g. monitored bed) and is most likely to have temporary (< 6 months) adverse effect on outcome.
4	Adverse event requires invasive (e.g. surgery) or complex treatment (e.g. monitored bed) and is most likely to have prolonged (≥ 6 months) adverse effect on outcome.
5	Sentinel or significant life or limb threatening event.
6	Adverse event resulting in death.

During the data collection period, trained research assistants regularly interviewed surgeons, residents, and nurse practitioners involved with orthopaedic and spine cases. For any reported adverse events, the research assistant asked the health care provider to classify its type and severity using the OrthoSAVES tool. Postoperative adverse events were collected up to discharge; this time frame included any time spent in other departments of the hospital during the surgical visit (e.g., the intensive care unit), but excluded any postoperative care and associated cost in other facilities (e.g., after transfer to an external hospital, rehab facility, or long-term care facility). After the data collection period was complete, the dataset was verified by a trained research assistant who reviewed charts for all admissions.

### Cost data

Hospital cost data were obtained from the hospital’s financial department, as part of the Ontario Case Costing Initiative (OCCI) [28]. The OCCI provides a standardized provincial methodology for calculating actual patient care cost. Our primary analysis considered total hospital costs, including both direct costs (e.g., OR and recovery room costs, implants/disposables, nursing and allied health, imaging costs, lab costs, etc.) and indirect costs (e.g., administrative- and facility-related overheads). All costs are from the perspective of the hospital, and do not include physician billings. Costs were inflated to 2021 values using Ontario-specific Consumer Price Index from Statistics Canada [29].

Costs separated by grouped functional centre (FC) were also considered as a set of secondary cost outcomes. The FC groups were nursing, operating room, intensive care unit (ICU), ambulatory care, pharmacy, patient food, respiratory therapy, allied health, and diagnostic/therapeutic services (see Additional File 2).

### Patient and admission data

To supplement the prospective OrthoSAVES data, each patient’s age, sex, body-mass index (BMI), American Society of Anesthesiologists (ASA) physical status, comorbid conditions, diagnosis category, procedure category, admission type (planned or unplanned), operating time, revision versus primary surgery, and length of stay were extracted via retrospective chart review. As operating time is known to have substantially different distributions in spine, hip, and knee surgery [24], it was categorized by tertile within each anatomical region.

Admission type was classified as planned or unplanned based on the priority level assigned to each case; these were determined by the consulting surgeon at decision to operate. Cases flagged as priority level 1 (surgery targeted within seven days from decision to operate) in the hospital’s operating room scheduling database were classified as “unplanned”, and all lower priority levels were classified as “planned”. Priority level 1 includes subcategories 1A (target 0–2 h), 1B (2–8 h), 1C (8–48 h), and 1D (2–7 days) [30]. These cases were either admitted through the hospital’s Emergency Department or were direct transfers from external institutions for urgent/emergent surgery.

### Statistical analysis

#### Multiple imputation

10% of records had incomplete data; the most commonly missing variable was body-mass index (8% missing). Unplanned and fracture-related admissions were more likely to have incomplete data (see comparison in Supplemental Table 2-A, Additional File 1). To address the issue, multiple imputation by chained equations [31] was used to generate 20 imputed datasets, assuming that missing values were missing at random. Imputation details are given in Sect. 2 of Additional File 1. Analysis results from each imputed dataset were pooled according to Rubin’s rules [31]. The imputed values compared reasonably to the observed values, so imputed results are presented.

#### Propensity-matched analysis

A propensity-matched analysis was used to determine the hospital cost and length of stay (LOS) attributable to AEs independent of other patient and surgical factors. This approach was selected over a regression approach to directly model cost and LOS because it provides absolute estimates of costs and bed-days independently attributable to AEs; in contrast, the regression approach would require transforming the cost and LOS variables to accommodate their non-negative, right-skewed distributions, and applying additional assumptions to derive absolute estimates (for example, using gamma regression with a log link function; see Sensitivity analysis below).

For our primary analysis, cases with AEs were matched to non-AE cases on propensity of having an AE. To determine propensity scores, multivariable logistic regression stratified by anatomical region was used to predict each patient’s risk of having an adverse event considering the following factors: age (65 + vs. <65 years); sex; BMI category (normal, overweight, obese class I/II, or obese class III); revision versus primary procedure; planned versus unplanned admission, pre-operative ASA grade 3 or higher; tertile of operating time; and number of reported comorbidities.

Each AE case was matched to two control cases using nearest-neighbor matching with replacement, with exact matching on anatomical region. A caliper of 0.2 standard deviations of the logit of the propensity score was specified [32]. Incremental cost and LOS for each matched set was calculated as the difference in cost/LOS between each AE case and the mean of its two matched controls. Paired t-tests were used to determine the statistical significance of the incremental values.

Incremental cost and LOS estimates were also calculated for high-severity versus low-severity AEs, using the matched cases and controls. As well, incremental cost and LOS were calculated for AEs in planned and unplanned (i.e., urgent/emergent) cases.

As a secondary analysis, incremental costs were also calculated for each grouped functional centre (see Additional File 2).

### Sensitivity analysis

To assess the robustness of the incremental cost/LOS estimates derived from propensity score matching, a sensitivity analysis was performed using gamma generalized linear modelling with a log link function to model both cost and length of stay (see details in Sect. 3 of Additional File 1).

## Results

### Sample description

For the prospective QI period, the OrthoSAVES database contained records on 3063 unique patients undergoing inpatient surgical procedures of the hip (N = 1154), knee (N = 1073), or spine (N = 836). Descriptive statistics for the overall sample and by AE occurrence are shown in Tables 2A (patient characteristics), 2B (admission and procedure characteristics) and 2C (adverse event rates).

**Table 2:** A. Patient characteristics, overall and by AE occurrence

Measure	Category	Overall (N = 3063)	Adverse events		p
			None (N = 2142)	One or more AEs (N = 921)	
Anatomical region	Spine	27.3% (836)	26.1% (558)	30.2% (278)	0.002
	Hip	37.7% (1154)	36.9% (790)	39.5% (364)	
	Knee	35.0% (1073)	37.1% (794)	30.3% (279)	
Sex	Male	44.9% (1374)	44.0% (943)	46.8% (431)	0.166
	Female	55.1% (1689)	56.0% (1199)	53.2% (490)	
Age (years)	Mean ± SD	64.1 ± 14.7 (N = 3063)	63.0 ± 14.5 (N = 2142)	66.6 ± 14.8 (N = 921)	< 0.001
Age group	< 65 years	48.3% (1479)	51.2% (1097)	41.5% (382)	< 0.001
	≥ 65 years	51.7% (1584)	48.8% (1045)	58.5% (539)	
Body-mass index (BMI, kg/m²)	Mean ± SD	29.3 ± 6.8 (N = 2824)	29.4 ± 6.8 (N = 1987)	29.0 ± 6.8 (N = 837)	0.219
BMI category	Underweight or normal (< 25 kg/m²)	26.1% (738)	25.2% (500)	28.4% (238)	0.058
	Overweight (25-29.9 kg/m²)	34.6% (976)	35.8% (712)	31.5% (264)	
	Obese class I/II (30-39.9 kg/m²)	32.5% (917)	31.9% (633)	33.9% (284)	
	Obese class III (≥ 40 kg/m²)	6.8% (193)	7.1% (142)	6.1% (51)	
	Not reported	— (239)	— (155)	— (84)	
Preoperative ASA physical status grade	1 (Healthy)	2.7% (82)	3.0% (65)	1.8% (17)	< 0.001
	2 (Mild systemic disease)	41.1% (1259)	43.8% (938)	34.9% (321)	
	3 (Severe systemic disease)	50.5% (1547)	48.7% (1043)	54.7% (504)	
	4 (Life-threatening systemic disease)	5.6% (172)	4.4% (94)	8.5% (78)	
	5 (Moribund, not expected to survive without operation)	0.1% (3)	0.1% (2)	0.1% (1)	
Preoperative ASA category	1–2	43.8% (1341)	46.8% (1003)	36.7% (338)	< 0.001
	≥ 3	56.2% (1722)	53.2% (1139)	63.3% (583)	
Comorbid conditions	Hypertension	50.5% (1547)	49.6% (1062)	52.7% (485)	0.124
	Asthma or COPD	16.3% (498)	16.5% (353)	15.7% (145)	0.631
	Diabetes	16.5% (504)	15.7% (337)	18.1% (167)	0.111
	Ulcer or stomach disorder	41.8% (1280)	41.5% (889)	42.5% (391)	0.632
	Kidney disease	11.7% (358)	11.0% (236)	13.2% (122)	0.086
	Liver disease	5.3% (162)	5.2% (111)	5.5% (51)	0.725
	Anemia or other blood disorder	10.5% (323)	10.6% (227)	10.4% (96)	0.949
	Cancer	8.8% (270)	8.6% (185)	9.2% (85)	0.627
	Depression	10.8% (331)	9.9% (212)	12.9% (119)	0.016
	Osteoarthritis	70.3% (2154)	71.4% (1530)	67.8% (624)	0.043
	Chronic back pain	39.6% (1213)	39.7% (850)	39.4% (363)	0.904
	Rheumatoid arthritis	3.5% (106)	3.5% (75)	3.4% (31)	0.914
	Coronary artery disease and/or history of heart attack	7.4% (227)	7.3% (157)	7.6% (70)	0.822
	History of heart failure	7.3% (225)	6.7% (143)	8.9% (82)	0.034
	History of stroke	5.4% (164)	4.4% (95)	7.5% (69)	< 0.001
	Hypercholesterolemia	29.3% (896)	28.1% (601)	32.0% (295)	0.027
Number of comorbid conditions	Mean ± SD (number of conditions)	3.3 ± 1.9 (N = 3063)	3.3 ± 1.9 (N = 2142)	3.5 ± 1.9 (N = 921)	0.016

Figures presented as mean ± standard deviation or percentage (count). Two-sample t-test or chi-square test of independence, as appropriate. AE = adverse event; ASA = American Society of Anaesthesiologists

**Table 2:** B. Admission and procedure characteristics, overall and by AE occurrence

Measure	Category	Overall (N = 3063)	Adverse events		p
			None (N = 2142)	One or more AEs (N = 921)	
Primary/revision procedure	Primary procedure	89.0% (2727)	90.2% (1933)	86.2% (794)	0.001
	Revision	11.0% (336)	9.8% (209)	13.8% (127)	
Procedure	Hip arthroplasty	30.2% (925)	30.9% (662)	28.6% (263)	< 0.001
	Hip reduction/fixation	6.9% (212)	5.5% (117)	10.3% (95)	
	Other hip procedure	0.6% (17)	0.5% (11)	0.7% (6)	
	Knee arthroplasty	33.1% (1015)	35.0% (749)	28.9% (266)	
	Knee arthroscopic procedure^a^	0.3% (10)	0.4% (8)	0.2% (2)	
	Knee ligament/tendon repair^a^	0.2% (5)	0.2% (4)	0.1% (1)	
	Knee reduction/fixation	1.0% (30)	1.2% (26)	0.4% (4)	
	Other knee procedure	0.4% (13)	0.3% (7)	0.7% (6)	
	Spine discectomy or decompression^a^	3.1% (96)	2.8% (60)	3.9% (36)	
	Spine fusion	20.5% (628)	19.8% (424)	22.1% (204)	
	Spine intradural procedure	1.0% (32)	1.3% (27)	0.5% (5)	
	Spine osteotomy procedure	1.7% (52)	1.3% (27)	2.7% (25)	
	Other spine procedure	0.9% (28)	0.9% (20)	0.9% (8)	
Admission type	Planned	79.5% (2435)	81.6% (1747)	74.7% (688)	< 0.001
	Unplanned	20.5% (628)	18.4% (395)	25.3% (233)	
Operating time: incision to close (minutes)	Mean ± SD	107.4 ± 86.3 (N = 3061)	100.6 ± 72.4 (N = 2141)	123.1 ± 110.6 (N = 920)	0.003
Operating time category	< 1 h	24.2% (742)	24.2% (519)	24.2% (223)	< 0.001
	1-1.9 h	49.8% (1524)	51.3% (1098)	46.3% (426)	
	2-2.9 h	10.7% (327)	11.8% (252)	8.2% (75)	
	3-3.9 h	7.7% (236)	7.3% (156)	8.7% (80)	
	4-4.9 h	3.6% (111)	3.2% (68)	4.7% (43)	
	≥ 5 h	4.0% (121)	2.2% (48)	7.9% (73)	
	Not reported	— (2)	— (1)	— (1)	
Anatomical region-specific tertile of operating time	Lower	33.2% (1015)	34.1% (731)	30.9% (284)	0.004
	Middle	32.0% (981)	33.0% (707)	29.8% (274)	
	Upper	34.8% (1065)	32.8% (703)	39.3% (362)	
	Not reported	— (2)	— (1)	— (1)	
Blood transfusion during admission	No	83.9% (2550)	88.7% (1884)	72.7% (666)	< 0.001
	Yes	16.1% (491)	11.3% (241)	27.3% (250)	
	Not reported	— (22)	— (17)	— (5)	
Discharge disposition	Transferred to other facility / other	35.0% (1073)	31.0% (665)	44.3% (408)	< 0.001
	Discharged home, with or without support services	65.0% (1990)	69.0% (1477)	55.7% (513)	
Length of admission	Mean ± SD (days)	7.3 ± 11.8 (N = 3063)	5.6 ± 5.6 (N = 2142)	11.1 ± 19.2 (N = 921)	< 0.001
Hospital admission cost (2021 $CAD)	Mean ± SD (CAD)	18202.7 ± 34944.6 (N = 3046)	14844.1 ± 25741.2 (N = 2129)	26000.3 ± 49324.2 (N = 917)	< 0.001

^a^Inpatient admissions were rare for soft tissue procedures such as knee arthroscopy, knee ligament/tendon repair, and spine discectomy; these exceptions were either non-ambulatory emergency cases or high-risk cases with significant comorbidities

Figures are presented as mean ± standard deviation or percentage (count). Two-sample t-test or chi-square test of independence, as appropriate. AE = adverse event

**Table 2:** C. Adverse event information, overall and by anatomical region

Measure	Category	All anatomical regions		Spine admissions		Hip admissions		Knee admissions	
		Overall (N = 3063)	One or more AEs (N = 921)	Overall (N = 836)	One or more AEs (N = 278)	Overall (N = 1154)	One or more AEs (N = 364)	Overall (N = 1073)	One or more AEs (N = 279)
Number of adverse events (AEs)	None	69.9% (2142)	—	66.7% (558)	—	68.5% (790)	—	74.0% (794)	—
	1	22.3% (683)	74.2% (683)	21.3% (178)	64.0% (178)	23.6% (272)	74.7% (272)	21.7% (233)	83.5% (233)
	2 or more	7.8% (238)	25.8% (238)	12.0% (100)	36.0% (100)	8.0% (92)	25.3% (92)	4.3% (46)	16.5% (46)
Any intraoperative AEs	No	96.8% (2966)	89.5% (824)	89.7% (750)	69.1% (192)	99.6% (1149)	98.6% (359)	99.4% (1067)	97.8% (273)
	Yes	3.2% (97)	10.5% (97)	10.3% (86)	30.9% (86)	0.4% (5)	1.4% (5)	0.6% (6)	2.2% (6)
Any postoperative AEs	No	72.0% (2206)	6.9% (64)	73.3% (613)	19.8% (55)	68.8% (794)	1.1% (4)	74.5% (799)	1.8% (5)
	Yes	28.0% (857)	93.1% (857)	26.7% (223)	80.2% (223)	31.2% (360)	98.9% (360)	25.5% (274)	98.2% (274)
Severity grade of worst AE	1	0.6% (19)	2.1% (19)	1.0% (8)	3.0% (8)	0.5% (6)	1.7% (6)	0.5% (5)	1.8% (5)
	2	23.8% (724)	79.9% (724)	22.4% (186)	68.6% (186)	27.0% (310)	86.6% (310)	21.3% (228)	82.3% (228)
	3	4.5% (138)	15.2% (138)	7.1% (59)	21.8% (59)	3.1% (36)	10.1% (36)	4.0% (43)	15.5% (43)
	4	0.4% (11)	1.2% (11)	1.2% (10)	3.7% (10)	0.0% (0)	0.0% (0)	0.1% (1)	0.4% (1)
	5	0.2% (6)	0.7% (6)	0.7% (6)	2.2% (6)	0.0% (0)	0.0% (0)	0.0% (0)	0.0% (0)
	6	0.3% (8)	0.9% (8)	0.2% (2)	0.7% (2)	0.5% (6)	1.7% (6)	0.0% (0)	0.0% (0)
	Not reported	— (15)	— (15)	— (7)	— (7)	— (6)	— (6)	— (2)	— (2)
Any low-severity AEs (grade < 3)	No	73.4% (2236)	10.4% (94)	71.7% (594)	13.3% (36)	70.7% (812)	6.1% (22)	77.5% (830)	13.0% (36)
	Yes	26.6% (812)	89.6% (812)	28.3% (235)	86.7% (235)	29.3% (336)	93.9% (336)	22.5% (241)	87.0% (241)
	Not reported	— (15)	— (15)	— (7)	— (7)	— (6)	— (6)	— (2)	— (2)
Any high-severity AEs (grade ≥ 3)	No	94.7% (2885)	82.0% (743)	90.7% (752)	71.6% (194)	96.3% (1106)	88.3% (316)	95.9% (1027)	84.1% (233)
	Yes	5.3% (163)	18.0% (163)	9.3% (77)	28.4% (77)	3.7% (42)	11.7% (42)	4.1% (44)	15.9% (44)
	Not reported	— (15)	— (15)	— (7)	— (7)	— (6)	— (6)	— (2)	— (2)

Figures presented as percentage (count). AE = adverse event

Overall, the sample was 45% male and mean age was 64.1 years (SD = 14.7 years), with 52% of the sample being age 65 or older (Table 2A). The mean body-mass index (BMI) was 29.3 kg/m^2^ (SD = 6.8 kg/m^2^), with 26% classified normal weight or underweight (BMI < 25), 35% overweight (BMI 25-29.9), 33% obese class I or II (BMI 30-39.9), and 7% obese class III (BMI ≥ 40). Preoperatively, most patients in the sample were categorized as ASA class 2 (41%, mild systemic disease) or 3 (51%, severe systemic disease). The most common comorbid conditions were osteoarthritis (70%), hypertension (51%), chronic back pain (40%), and hypercholesterolemia (29%); patients had 3.3 comorbid conditions on average (SD = 1.9). Spine and hip procedures, older age, non-degenerative diagnosis, preoperative ASA grade of 3 or higher, and more comorbid conditions were each significantly associated with AE risk in bivariate analysis; higher body-mass index category was marginally significantly associated with AE risk.

11% of admissions in the cohort were for revision procedures (Table 2B). The most common procedures in the sample were knee arthroplasty (33%), hip arthroplasty (30%), spine fusion (21%), and hip reduction and fixation (7%). Unplanned admissions accounted for 628 cases (21% of the sample); the majority of unplanned cases were priority 1C (N = 455; surgery targeted within 8–48 h from decision to operate) or priority 1B (N = 146, target 2–8 h from decision to operate). The most common unplanned procedures were hip reduction and fixation (N = 209), spine fusion (N = 163), and hip arthroplasty (N = 141). Mean operating time from incision to close was 107 min (SD = 86 min), with 50% of cases being 1 to 2 h long. Patients received blood transfusions in 16% of admissions. 35% of patients were not discharged home (i.e., transferred to another facility, deceased, or signed out against medical advice). In bivariate analysis, AEs were more likely with revision cases, hip reduction/fixation, spine fusion, unplanned admissions, longer operating time, receiving a blood transfusion, and not being discharged home.

Average case cost was $18,203 CAD (SD = $34,945, Table 2B) and average length of stay was 7.3 days (SD = 11.8); both were significantly greater in cases with AEs (p < 0.001). The median length of stay was 4 days, and 98% of patients were discharged in fewer than 30 days.

Overall, 30% of cases had at least one intra- or postoperative AE (Table 2C); the rates were 33%, 31% and 26% for spine, hip, and knee cases respectively. Intraoperative AEs occurred in 3% of cases, while postoperative AEs occurred in 28% of cases. Among cases with AEs, 26% had more than one event during the admission; 82% had a low-severity event at worst (grade 1–2) and 18% had at least one high-severity event (grade 3–6).

The most common intra-operative AE was dural tear; the most commonly reported post-operative AEs included urinary retention, urinary tract infection, delirium, cardiac events (e.g. arrhythmia, heart failure), pulmonary embolism, and deep wound infection (Table 3).

**Table 3:** Most common adverse events, overall and by case type

Rank		All admissions (n = 3063)		Planned cases (n = 2435)		Unplanned cases (n = 628)	
		Category	% (count)	Category	% (count)	Category	% (count)
**Intra-operative adverse events**							
	**—**	**All categories**	**3.2% (97)**	**All categories**	**3.6% (88)**	**All categories**	**1.4% (9)**
	**1**	Dural tear	2.0% (62)	Dural tear	2.4% (59)	Dural tear	0.5% (3)
	**2**	Other intra-operative AE	0.7% (20)	Other intra-operative AE	0.7% (18)	Other intra-operative AE	0.3% (2)
	**3**	Massive blood loss (> 5 L in 24 h or > 2 L in 3 h)	0.2% (7)	Massive blood loss (> 5 L in 24 h or > 2 L in 3 h)	0.2% (6)	Massive blood loss (> 5 L in 24 h or > 2 L in 3 h)	0.2% (1)
	**4**	Neural injury – spinal cord	0.1% (3)	Neural injury – spinal cord	0.1% (3)	Neural injury – nerve root	0.2% (1)
	**5**	Neural injury – nerve root	0.1% (2)	Hardware malpositioning requiring revision	0.1% (2)	Cardiac event	0.2% (1)
**Post-operative adverse events**							
	**—**	**All categories**	**28.0% (857)**	**All categories**	**25.8% (628)**	**All categories**	**36.5% (229)**
	**1**	Urinary retention	10.7% (327)	Urinary retention	12.2% (296)	Other post-operative AE^1^	12.9% (81)
	**2**	Other post-operative AE^1^	7.5% (231)	Other post-operative AE^1^	6.2% (150)	Urinary tract infection	11.8% (74)
	**3**	Urinary tract infection	4.1% (126)	Urinary tract infection	2.1% (52)	Delirium/altered mental status	6.8% (43)
	**4**	Delirium/ altered mental status	2.6% (81)	Delirium/altered mental status	1.6% (38)	Cardiac arrythmia/failure/ arrest	5.6% (35)
	**5**	Cardiac arrythmia/ failure/arrest	2.3% (71)	Cardiac arrythmia/ failure/arrest	1.5% (36)	Urinary retention	4.9% (31)
	**6**	Pulmonary embolism	1.4% (42)	Pulmonary embolism	1.2% (30)	Pneumonia	3.5% (22)
	**7**	Deep wound infection	1.2% (36)	Deep wound infection	1.1% (26)	Pulmonary embolism	1.9% (12)
	**8**	Pneumonia	1.1% (33)	Superficial wound infection	0.9% (23)	Airway/breathing	1.8% (11)
	**9**	Superficial wound infection	1.0% (31)	Airway/breathing	0.7% (17)	Ileus/bowel obstruction	1.6% (10)
	**10**	Airway/ breathing	0.9% (28)	Fall	0.6% (14)	Deep wound infection	1.6% (10)

^1^Most common “other” post-operative AEs (counts): “Blood transfusion” (105), “Revision” (36), “C-diff” (14), “Painful hardware” (14), “Event not specified” (11), “Hyponatremia” (11)

On average, admissions with AEs were $11,100 more costly (95% CI: $8,400-$13,700) and 5.5 days longer (95% CI: 4.6–6.4 days) than admissions without AEs (Table 4). The differences were larger in unplanned admissions, among which admissions with AEs were $19,100 more costly (95% CI: $13,400-$24,800) and 10.1 days longer (95% CI: 7.6–12.7 days) on average.

**Table 4:** Unadjusted mean cost and length of stay by AE status

Group	Case type	Severity of worst AE	AE % (cases/N)	Hospital case cost, thousands of dollars Mean ± SD (95% CI)			Length of stay: days Mean ± SD (95% CI)		
				No AEs	One or more AEs	Unadjusted difference	No AEs	One or more AEs	Unadjusted difference
All anatomical regions	All	Any	30% (921/3063)	14.9 ± 25.7 (13.8, 16.0)	25.9 ± 49.2 (22.8, 29.1)	11.1 ± 34.5 (8.4, 13.7)	5.6 ± 5.6 (5.4, 5.8)	11.1 ± 19.2 (9.9, 12.4)	5.5 ± 11.5 (4.6, 6.4)
		Low	26% (756/2898)	14.9 ± 25.7 (13.8, 16.0)	20.8 ± 21.1 (19.3, 22.3)	5.9 ± 24.6 (3.9, 7.9)	5.6 ± 5.6 (5.4, 5.8)	9.5 ± 10.8 (8.7, 10.2)	3.9 ± 7.3 (3.3, 4.5)
		High	7% (165/2307)	14.9 ± 25.7 (13.8, 16.0)	49.9 ± 104.5 (34.0, 65.8)	35.0 ± 37.3 (29.1, 40.9)	5.6 ± 5.6 (5.4, 5.8)	18.7 ± 38.5 (12.8, 24.6)	13.1 ± 11.6 (11.3, 14.9)
	Planned	Any	28% (688/2435)	13.4 ± 26.9 (12.1, 14.6)	21.0 ± 47.1 (17.5, 24.5)	7.6 ± 33.9 (4.6, 10.6)	4.5 ± 2.9 (4.4, 4.6)	7.9 ± 16.9 (6.6, 9.1)	3.4 ± 9.3 (2.6, 4.2)
		Low	25% (574/2321)	13.4 ± 26.9 (12.1, 14.6)	17.1 ± 15.4 (15.9, 18.4)	3.7 ± 24.6 (1.4, 6.1)	4.5 ± 2.9 (4.4, 4.6)	6.6 ± 6.0 (6.1, 7.1)	2.1 ± 3.9 (1.7, 2.5)
		High	6% (114/1861)	13.4 ± 26.9 (12.1, 14.6)	40.8 ± 109.2 (20.7, 60.8)	27.4 ± 37.5 (20.3, 34.5)	4.5 ± 2.9 (4.4, 4.6)	14.5 ± 39.1 (7.4, 21.7)	10.1 ± 10.0 (8.2, 12.0)
	Unplanned	Any	37% (233/628)	21.5 ± 17.9 (19.7, 23.3)	40.6 ± 52.6 (33.8, 47.3)	19.1 ± 35.0 (13.4, 24.8)	10.6 ± 10.2 (9.6, 11.6)	20.7 ± 22.2 (17.8, 23.6)	10.1 ± 15.8 (7.6, 12.7)
		Low	32% (182/577)	21.5 ± 17.9 (19.7, 23.3)	32.3 ± 30.6 (27.9, 36.8)	10.8 ± 22.7 (6.9, 14.8)	10.6 ± 10.2 (9.6, 11.6)	18.7 ± 16.1 (16.3, 21.0)	8.1 ± 12.3 (5.9, 10.3)
		High	11% (51/446)	21.5 ± 17.9 (19.7, 23.3)	70.0 ± 91.0 (45.0, 94.9)	48.5 ± 34.9 (38.3, 58.7)	10.6 ± 10.2 (9.6, 11.6)	27.9 ± 35.9 (18.1, 37.7)	17.3 ± 15.4 (12.8, 21.8)
Hip	All	Any	32% (364/1154)	12.5 ± 9.7 (11.8, 13.2)	19.2 ± 20.5 (17.1, 21.3)	6.7 ± 14.0 (5.0, 8.5)	5.7 ± 7.2 (5.2, 6.2)	11.0 ± 15.0 (9.4, 12.5)	5.2 ± 10.3 (3.9, 6.5)
	Planned	Any	27% (210/792)	10.4 ± 2.7 (10.2, 10.7)	12.2 ± 5.1 (11.6, 12.9)	1.8 ± 3.5 (1.2, 2.3)	3.8 ± 1.5 (3.7, 4.0)	5.2 ± 3.5 (4.7, 5.6)	1.3 ± 2.2 (1.0, 1.7)
	Unplanned	Any	43% (154/362)	18.2 ± 17.1 (15.9, 20.6)	28.7 ± 28.4 (24.3, 33.2)	10.5 ± 22.6 (5.8, 15.2)	11.1 ± 12.3 (9.4, 12.7)	18.8 ± 20.2 (15.6, 22.0)	7.8 ± 16.1 (4.4, 11.1)
Knee	All	Any	26% (279/1073)	10.2 ± 3.2 (10.0, 10.5)	12.2 ± 6.8 (11.4, 13.0)	1.9 ± 4.4 (1.3, 2.5)	4.1 ± 1.8 (4.0, 4.3)	5.6 ± 4.5 (5.1, 6.2)	1.5 ± 2.8 (1.1, 1.9)
	Planned	Any	26% (266/1020)	10.1 ± 2.4 (10.0, 10.3)	12.0 ± 6.5 (11.2, 12.8)	1.9 ± 3.9 (1.3, 2.4)	4.0 ± 1.3 (3.9, 4.1)	5.4 ± 4.1 (4.9, 5.9)	1.4 ± 2.4 (1.1, 1.7)
	Unplanned	Any	25% (13/53)	12.1 ± 9.7 (9.1, 15.1)	15.3 ± 10.8 (9.4, 21.2)	3.2 ± 9.9 (-3.0, 9.4)	6.5 ± 5.4 (4.8, 8.2)	10.2 ± 8.7 (5.4, 14.9)	3.7 ± 6.4 (-0.3, 7.6)
Spine	All	Any	33% (278/836)	24.8 ± 47.5 (20.9, 28.8)	48.6 ± 81.8 (39.0, 58.2)	23.7 ± 61.0 (15.0, 32.5)	7.5 ± 6.1 (7.0, 8.0)	16.9 ± 29.2 (13.4, 20.3)	9.3 ± 17.5 (6.8, 11.9)
	Planned	Any	34% (212/623)	23.5 ± 54.2 (18.2, 28.7)	40.9 ± 81.0 (30.0, 51.8)	17.4 ± 64.5 (6.7, 28.1)	6.3 ± 5.0 (5.8, 6.8)	13.7 ± 29.2 (9.7, 17.6)	7.4 ± 17.5 (4.5, 10.3)
	Unplanned	Any	31% (66/213)	28.7 ± 18.4 (25.7, 31.6)	73.2 ± 80.0 (53.9, 92.5)	44.6 ± 47.0 (30.9, 58.2)	11.0 ± 7.3 (9.8, 12.2)	27.1 ± 26.8 (20.7, 33.6)	16.1 ± 16.1 (11.4, 20.8)

AE = adverse event; SD = standard deviation; CI = confidence interval

Cost data separated by grouped functional centre were available for almost all admissions in the cohort (N = 3050). Secondary analysis showed that the cost differences between AE and non-AE cases were largest for the nursing and ICU functional centres (see Supplemental Table 5 A, Additional File 2).

### Incremental cost and bed-days attributable to adverse events

The number of cases successfully matched to two controls ranged from 914 to 920 across imputations (99.2–99.9% of the 921 cases). The full propensity models for each anatomical region are shown in Sect. 4 of Additional File 1. Sensitivity analysis using gamma-log regression to estimate the cost and LOS attributable to AEs provided comparable, though slightly more conservative estimates (full results in Sect. 3, Additional File 1).

### Incremental costs

Cost estimates are given in Table 5. The estimated incremental cost attributable to AEs was $8,500 per admission (95% CI: $5,100 − 11,800). Cumulatively, the estimated total cost attributable to AEs in the cohort was $7.8 million, or 14.0% of all hospital expenditures for this cohort (95% CI: 8.5–19.6%).

**Table 5:** Hospital cost attributable to AEs from propensity-matched analysis

Group	Case type	Severity of worst AE (versus no AEs)	AE % (95% CI)	Incremental cost per case with AEs, $ thousands (95% CI)	Cumulative cost attributable to AEs, $ millions (95% CI)^a^	% of subgroup cost attributable to AEs (95% CI)^b^	% of total cohort cost attributable to AEs (95% CI)^c^
All anatomical regions	All (N = 3063)	Any	30.1 (28.4, 31.7)	8.5 (5.1, 11.8)	7.82 (4.73, 10.90)	—	14.0 (8.5, 19.6)
		Low	24.7 (23.2, 26.2)	4.0 (2.0, 5.9)	3.00 (1.55, 4.45)	—	5.4 (2.8, 8.0)
		High	5.3 (4.6, 6.1)	29.5 (13.1, 45.8)	4.83 (2.15, 7.50)	—	8.7 (3.9, 13.5)
	Planned (N = 2435)	Any	28.3 (26.5, 30.0)	4.7 (0.9, 8.5)	3.23 (0.65, 5.81)	8.5 (1.7, 15.4)	5.8 (1.2, 10.4)
		Low	23.6 (21.9, 25.3)	1.6 (-0.3, 3.5)	0.91 (-0.17, 2.00)	2.4 (-0.5, 5.3)	1.6 (-0.3, 3.6)
		High	4.6 (3.8, 5.5)	20.7 (-0.1, 41.4)	2.33 (-0.01, 4.67)	6.2 (-0.0, 12.3)	4.2 (-0.0, 8.4)
	Unplanned (N = 628)	Any	37.1 (33.3, 40.9)	19.8 (12.7, 26.8)	4.61 (2.96, 6.25)	25.7 (16.5, 34.8)	8.3 (5.3, 11.2)
		Low	29.0 (25.4, 32.5)	11.6 (6.4, 16.7)	2.10 (1.16, 3.04)	11.7 (6.5, 17.0)	3.8 (2.1, 5.5)
		High	8.1 (6.0, 10.3)	48.8 (23.9, 73.7)	2.50 (1.22, 3.77)	13.9 (6.8, 21.0)	4.5 (2.2, 6.8)
Hip	All (N = 1154)	Any	31.5 (28.9, 34.2)	4.9 (2.7, 7.1)	1.79 (0.98, 2.59)	10.6 (5.8, 15.4)	3.2 (1.8, 4.7)
	Planned (N = 792)	Any	26.5 (23.4, 29.6)	1.0 (0.1, 1.9)	0.21 (0.02, 0.39)	2.4 (0.3, 4.5)	0.4 (0.0, 0.7)
	Unplanned (N = 362)	Any	42.5 (37.4, 47.6)	10.3 (5.3, 15.4)	1.59 (0.82, 2.36)	19.4 (10.0, 28.8)	2.9 (1.5, 4.2)
Knee	All (N = 1073)	Any	26.0 (23.4, 28.6)	1.9 (1.1, 2.7)	0.53 (0.30, 0.76)	4.6 (2.6, 6.6)	1.0 (0.5, 1.4)
	Planned (N = 1020)	Any	26.1 (23.4, 28.8)	1.8 (1.0, 2.6)	0.47 (0.26, 0.68)	4.3 (2.4, 6.3)	0.8 (0.5, 1.2)
	Unplanned (N = 53)	Any	24.5 (12.9, 36.1)	4.7 (-1.5, 10.9)	0.06 (-0.02, 0.14)	9.0 (-2.8, 20.7)	0.1 (-0.0, 0.3)
Spine	All (N = 836)	Any	33.3 (30.1, 36.4)	19.8 (9.2, 30.4)	5.51 (2.56, 8.45)	20.1 (9.4, 30.9)	9.9 (4.6, 15.2)
	Planned (N = 623)	Any	34.0 (30.3, 37.7)	12.1 (-0.0, 24.2)	2.56 (-0.01, 5.13)	14.0 (-0.0, 28.0)	4.6 (-0.0, 9.2)
	Unplanned (N = 213)	Any	31.0 (24.8, 37.2)	44.5 (23.7, 65.3)	2.94 (1.56, 4.31)	32.5 (17.3, 47.6)	5.3 (2.8, 7.7)

^a^ Cumulative cost attributable to AEs = (incremental cost per case) × (number of cases with AEs of the specified severity within the given subgroup of anatomical region and case type)

^b^ % of subgroup cost = (cumulative cost attributable to AEs) / (total cost for admissions in the anatomical region and case type subgroup)

^c^ % of total cohort cost = (cumulative cost attributable to AEs) / (total cost for all admissions, approximately $55.5 million)

AE = adverse event; CI = confidence interval

Hospital spending attributable to AEs varied with severity: low-severity AEs accounted for incremental cost of $4,000 per admission (95% CI: $2,000–5,900), while high-severity AEs accounted for $29,500 per admission (95% CI: $13,100 − 45,800). The cumulative spending attributable to low-severity AEs was $3.0 million (5.4% of all expenditures), while the cumulative spending attributable to high-severity AEs was $4.8 million (8.7% of expenditures).

Compared to planned admissions, adverse events in unplanned admissions were associated with considerably higher hospital spending. Among planned admissions (79% of the cohort), adverse events were associated with $4,700 higher admission cost (95% CI: $900-8,500), corresponding to 5.8% of all spending (95% CI: 1.2–10.4%). In contrast, among unplanned admissions (21% of the cohort), AEs were associated $19,800 higher admission cost (95% CI: $12,700-$26,800), which corresponded to 8.3% (95% CI: 5.3–11.2%) of all spending.

The incremental cost associated with high-severity AEs was particularly large in unplanned admissions, in which they accounted for an increase of $48,800 (95% CI: $23,900 − 73,700) per admission, corresponding to 4.5% of spending (95% CI: 2.2–6.8%) across the entire cohort.

Comparing anatomical regions, hospital spending attributable to AEs was substantially higher among spine surgery admissions ($19,800 [95% CI: $9,200 − 30,400] per admission) compared to hip ($4,900 [95% CI: $2,700-7,100]) or knee ($1,900 [95% CI: $1,100-$2,700]) admissions.

Incremental cost estimates separated by grouped functional centre are given in Supplemental Table 5B, Additional File 2. These results indicate that the increased costs associated with adverse events are largely driven by nursing and ICU costs. Increased nursing costs attributable to AEs accounted for 4.1% of spending in the cohort, and increased ICU costs accounted for 5.2% of spending. Costs attributable to AEs in these two functional centre groups were particularly concentrated among unplanned admissions and spine admissions.

### Incremental bed-days

Length of stay estimates are given in Table 6. The estimated LOS attributable to AEs was 4.7 days per admission (95% CI: 3.4–5.9). Cumulatively, this corresponded to a total of 4,290 bed-days across the cohort—19.3% of all bed-days (95% CI: 14.2–24.4%).

**Table 6:** Length of stay attributable to AEs from propensity-matched analysis

Group	Case type	Severity of worst AE (versus no AEs)	AE % (95% CI)	Incremental bed-days per case with AEs (95% CI)	Cumulative bed-days attributable to AEs, thousands (95% CI)^a^	% of subgroup bed-days attributable to AEs (95% CI)^b^	% of total cohort bed-days attributable to AEs (95% CI)^c^
All anatomical regions	All (N = 3063)	Any	30.1 (28.4, 31.7)	4.7 (3.4, 5.9)	4.29 (3.16, 5.43)	—	19.3 (14.2, 24.4)
		Low	24.7 (23.2, 26.2)	3.1 (2.3, 3.9)	2.34 (1.76, 2.92)	—	10.5 (7.9, 13.1)
		High	5.3 (4.6, 6.1)	11.9 (6.1, 17.8)	1.96 (0.99, 2.92)	—	8.8 (4.5, 13.1)
	Planned (N = 2435)	Any	28.3 (26.5, 30.0)	2.4 (1.1, 3.6)	1.63 (0.76, 2.50)	12.3 (5.7, 18.9)	7.3 (3.4, 11.2)
		Low	23.6 (21.9, 25.3)	1.2 (0.7, 1.7)	0.69 (0.39, 0.99)	5.2 (2.9, 7.5)	3.1 (1.7, 4.5)
		High	4.6 (3.8, 5.5)	8.4 (1.2, 15.6)	0.94 (0.13, 1.76)	7.1 (1.0, 13.2)	4.2 (0.6, 7.9)
	Unplanned (N = 628)	Any	37.1 (33.3, 40.9)	11.5 (8.5, 14.5)	2.68 (1.98, 3.37)	29.7 (22.0, 37.5)	12.0 (8.9, 15.2)
		Low	29.0 (25.4, 32.5)	9.1 (6.6, 11.7)	1.66 (1.20, 2.13)	18.5 (13.3, 23.6)	7.5 (5.4, 9.5)
		High	8.1 (6.0, 10.3)	19.8 (9.9, 29.7)	1.01 (0.50, 1.52)	11.2 (5.6, 16.9)	4.5 (2.3, 6.8)
Hip	All (N = 1154)	Any	31.5 (28.9, 34.2)	3.8 (2.3, 5.4)	1.39 (0.83, 1.95)	16.3 (9.7, 22.9)	6.2 (3.7, 8.8)
	Planned (N = 792)	Any	26.5 (23.4, 29.6)	0.2 (-0.4, 0.9)	0.05 (-0.09, 0.19)	1.6 (-2.7, 5.8)	0.2 (-0.4, 0.9)
	Unplanned (N = 362)	Any	42.5 (37.4, 47.6)	8.8 (5.4, 12.2)	1.35 (0.82, 1.88)	25.9 (15.8, 36.1)	6.1 (3.7, 8.4)
Knee	All (N = 1073)	Any	26.0 (23.4, 28.6)	1.5 (0.9, 2.0)	0.41 (0.26, 0.57)	8.5 (5.3, 11.7)	1.9 (1.2, 2.6)
	Planned (N = 1020)	Any	26.1 (23.4, 28.8)	1.3 (0.7, 1.8)	0.33 (0.20, 0.47)	7.5 (4.4, 10.6)	1.5 (0.9, 2.1)
	Unplanned (N = 53)	Any	24.5 (12.9, 36.1)	6.2 (1.4, 11.0)	0.08 (0.02, 0.14)	20.4 (4.6, 36.3)	0.4 (0.1, 0.6)
Spine	All (N = 836)	Any	33.3 (30.1, 36.4)	9.0 (5.5, 12.4)	2.49 (1.53, 3.46)	28.1 (17.2, 38.9)	11.2 (6.9, 15.5)
	Planned (N = 623)	Any	34.0 (30.3, 37.7)	5.9 (1.9, 9.9)	1.25 (0.41, 2.09)	22.8 (7.4, 38.3)	5.6 (1.8, 9.4)
	Unplanned (N = 213)	Any	31.0 (24.8, 37.2)	18.8 (12.2, 25.4)	1.24 (0.81, 1.68)	36.4 (23.7, 49.2)	5.6 (3.6, 7.5)

^a^ Cumulative bed-days attributable to AEs = (incremental bed-days per case) × (number of cases with AEs of the specified severity within the given subgroup of anatomical region and case type)

^b^ % of subgroup bed-days = (cumulative bed-days attributable to AEs) / (total bed-days for admissions in the anatomical region and case type subgroup)

^c^ % of total cohort cost = (cumulative bed-days attributable to AEs) / (total bed-days for all admissions, approximately 22,200 bed-days)

AE = adverse event; CI = confidence interval

Like costs, the length of stay associated with AEs varied greatly with AE severity: incremental length of stay was 3.1 days per admission (95% CI: 2.3–3.9) for low-severity AEs versus 11.9 days (95% CI: 6.1–17.8) per admission for high-severity AEs. However, unlike costs, low- and high-severity AEs cumulatively accounted for similar numbers of bed-days: 2,340 bed-days (10.5% of all bed-days) for low-severity AEs, versus 1,960 bed-days (8.8%) for high-severity AEs.

Considerably more bed-days were attributable to AEs in unplanned admissions compared to planned admissions, both per admission and in aggregate. Among planned admissions, AEs accounted for 2.4 additional bed-days per admission (95% CI: 1.1–3.6) and 7.3% of all bed-days (95% CI: 3.4–11.2%) in the cohort. Among unplanned admissions, AEs accounted for 11.5 additional bed-day days per admission (95% CI: 8.5–14.5) and 12.0% (95% CI: 8.9–15.2%) of all bed-days in the cohort.

High-severity AEs in unplanned admissions were associated with a particularly large number of bed-days, accounting for an additional 19.8 bed-days (95% CI: 9.9–29.7) per admission. This corresponded to 4.5% of all bed-days (95% CI: 2.3–6.8%) across the cohort.

As with costs, more bed-days were attributable to AEs in spine admissions compared to hip and knee admissions. Adverse events in spine admissions were associated with 9.0 more bed-days per admission [95% CI: 5.5–12.4] per admission), compared to 3.8 days [95% CI: 2.3–5.4] for hip admissions and 1.5 days [95% CI: 0.9-2.0] for knee admissions.

## Discussion

In the context of limited funding and an aging population, the sustainability of increasing volumes of orthopaedic and spine procedures is unknown. Our study represents a comprehensive analysis of hospital resource use attributable to AEs in inpatient orthopaedic and spine surgical admissions. Overall, 14% of hospital spending and 19% of all bed-days among this patient population can be attributed to AEs. Compared to low-severity AEs, high-severity AEs were associated with considerably larger per-admission increases in cost and LOS; however, cumulatively, the more common low-severity AEs accounted for 38% of costs ($3 million of $7.82 million) and 54% of bed-days (2,340 of 4,290 bed-days) attributable to AEs. Adverse events were more frequent among unplanned admissions, and their association with cost and LOS per unplanned admission was almost 5 times their association in planned admissions. Despite only 21% of admissions being unplanned, AEs in these admissions cumulatively accounted for 59% of costs ($4.61 million of $7.82 million) and 62% of bed-days (2,680 of 4,290 bed-days) attributable to AEs. Compared to the hip and knee groups, spine surgery AEs were associated with the highest incremental cost and LOS, both per-admission and cumulatively. Our secondary analysis of hospital costs separated by grouped functional centre showed that the hospital costs attributable to AEs are largely driven by nursing and ICU costs, and that these costs are particularly concentrated in unplanned admissions and spine admissions.

Our finding that AEs are associated with an additional $8,500 per admission is consistent with the Canadian Patient Safety Institute report of AE costs ranging from 4,028 to 12,648 CAD per case [8]. We report that individuals who experienced any AE had 4.7 days longer length of stay per admission, in line with previous reports that found 6 additional acute care days attributable to each AE [8, 9]. Longer admissions affect not only cost, but also patient safety. Hauck et al. [33] found that hospital stays carried inherent risks of adverse drug reactions, infections, and ulcers, and that these risks grew with increasing length of stay. In our cohort, 26% of patients with adverse events had multiple events, wherein the subsequent events often occurred as sequelae of the first (e.g., urinary tract infection due to prolonged indwelling urinary catheter to treat post-operative heart failure).

Our stratified findings are unique in that we reported the association of AEs with cost and LOS by severity grade of the worst AE. We categorized the severity grades into low (grade 1–2) and high (grade 3+) and found that most admissions with AEs had only low severity events (n = 756, 82% of admissions with AEs). Consistent with Rutberg et al. [34], the most common postoperative AEs were urinary retention, urinary tract infection, delirium, cardiac events, pulmonary embolism, and deep wound infection. Of these, urinary retention (n = 327) was the most common adverse event. Notably, the majority of low-severity AEs in our cohort are considered preventable [35] and, being among the most frequent AEs reported, are likely amenable to system-level mitigation strategies.

Another unique aspect of our study relates to the gross difference in frequency and cost of AEs between planned and unplanned admissions, where there is a paucity of comparative studies. The higher frequency of adverse events in unplanned cases was consistent with that of Sathiyakumar et al. [36] who used NSQIP data from 146,773 orthopaedic cases to demonstrate that the AE rate in trauma cases was nearly triple that of general orthopaedic patients. In our cohort, the cost attributable to adverse events in unplanned admissions was five times their cost in planned admissions (19,800 versus 4,700 CAD per admission, respectively). Although not directly comparable, to our knowledge the only other economic study assessing this subgroup was a population-based administrative data study by Tessier et al. [11], who found that the incremental cost of AEs (defined as preventable harm) was 2.2 times as high for unplanned versus planned surgical admissions. While the volume of unplanned urgent/emergent admissions is relatively low, the high cost of AEs in these cases requires further investigation, particularly from the perspective of preventative strategies [37]. The stratified findings of this study strengthen the economic argument to implement subgroup-specific strategies to improve patient safety. With AEs posing a significant burden on the healthcare system, and many AEs considered modifiable, our specialty-specific findings should be taken into account when developing broader patient safety initiatives. Economic reality dictates that quality improvement initiatives are more likely to be funded in areas where a compelling case exists for patient benefit and financial return on investment [7, 38]. For example, using evidence from this quality initiative as justification, our institution has implemented routine geriatric consultation for all non-ambulatory elderly unplanned cases, to facilitate perioperative metabolic and pharmacological optimization and reduce the risk of perioperative delirium [39]. Also, to reduce the risk of surgical site infection in unplanned cases when surgery is scheduled more than 24 h from admission, we have introduced surgical site chlorohexidine wash(es) as part of the preoperative process on the inpatient ward.

The bulk of past studies on the cost of AEs in orthopaedic surgery have looked at specific procedures, have inconsistent costing data, and have varying or unspecified definitions of AEs [17, 19, 40–45]. Within this context, this study has several notable strengths. First, the use of the validated OrthoSAVES tool enabled us to consistently capture and categorize the type and clinical severity of adverse events relevant to orthopaedic procedures compared to administrative data which historically underreports AEs [13–15, 17, 18, 40]. The implementation of NSQIP has enabled a tremendous advancement in collection and mitigation of AEs [46]. However, there exists significant variation in reported AE rates for common orthopaedic procedures amongst national registries [18], and as noted by Molina et al. [23], variables specific to orthopaedic surgery are needed within the NSQIP registry to more accurately reflect AE rates in this patient group. Additionally, Street et al. [13] demonstrated that compared to using ICD-10 administrative data, prospective use of SAVES resulted in a higher AE capture rate and provided appropriate breadth of specific AEs in a sub-specialty spine population. From a resource utilization and economic perspective, underreporting of AEs likely leads to an underestimate of their true association with cost and bed-days by having a greater portion of false negative cases within the non-AE cohort.

Second, we classified AEs by severity grades based on defined patient or process outcomes, rather than the more typical practice of categorizing AEs into undefined binary “minor” and “major” events [47, 48], or omitting severity classification entirely. A recent study by Chen et al. [26] comparing AE reporting by surgeons and independent clinical reviewers using SAVES and OrthoSAVES found that independent reviewers reported more low-severity AEs than surgeons. Our study highlights the economic and resource implication of underreporting “minor” AEs: we found that low-grade severity AEs occurred frequently, with substantial aggregate costs corresponding to 5.3% of all expenditures and 10.5% of all bed-days.

Third, we used patient-level data on AEs, costs, and a variety of relevant covariates. This enabled us to provide a more accurate estimate of incremental cost per admission, instead of the more common reporting of unadjusted mean cost in economic studies. Hellsten et al. [7] reported that the outcome of an economic evaluation can be influenced by the accuracy of patient data, costing data, and analysis used. Independent risk factors should also be considered as potential confounders when conducting an economic evaluation, as Millstone et al. [24] reported that increasing age, male sex, revision surgery, and increasing operative duration are associated with a higher likelihood of experiencing an adverse event. These independent risk factors can distort the relationship between cost and AEs. We analytically controlled for these factors with our propensity matching approach; however, other unknown confounders may still potentially bias the estimates.

Our analysis also has some limitations. One potential confounder we were unable to consider was patient race/ethnicity, which is not documented in clinical records at our institution. We believe the racial/ethnic distribution is comparable to that of other studies in similar settings; for example. a survey study of ambulatory patients in the same institution seeking consultation for elective orthopaedic treatment had a distribution of 78% White, 5% East Asian, 4.5% Black, 4.3% South Asian, and 7.8% other ethnicity [49]. A number of American studies have demonstrated that racial/ethnic minority patients have higher risk of adverse events for both planned and unplanned orthopaedic surgery [50–54]. Corresponding evidence in the Canadian orthopaedic population is lacking, though there is evidence that Indigenous Canadians have increased rates of postoperative complications when undergoing a variety of surgical procedures [55, 56]. Future Canadian studies on AEs in orthopaedic surgery should collect patient-level race/ethnicity data in order to quantify the relationship between race/ethnicity, AE risk, and hospital resource use in the Canadian context.

We did not include medication-related AEs, which are independently captured by our institution and not included in OrthoSAVES. This may have led to an underestimate of total AE-associated cost. We also did not capture postoperative AEs that occurred after discharge from our institution; this may have resulted in an undercount of AEs, particularly for the approximately 35% of patients who were transferred to an external hospital, rehab facility, or long-term care facility at the end of their admission.

As well, it is possible that a large proportion of the cost attributable to AEs is directly related to LOS. Our study period was 2011 to 2012, which was likely too early to reflect the ongoing trend of decreasing LOS for most planned orthopaedic procedures [57–59]. The majority of planned hip and knee replacement admissions in our institution are now 1 or 2 days long, versus 4 to 5 days at the time of study cohort. Average LOS for unplanned admissions and spine surgery admissions, however, remain stable. For simple admissions in our cohort (i.e., with no adverse events or ICU admission), the median cost per bed-day excluding operating room costs was $1,372 (interquartile range: $1,187-$1,588); this includes both direct costs of care and indirect overhead costs such as housekeeping, maintenance, and administrative support. Our analysis suggests that the average incremental cost per bed-day attributable to AEs is $1,808 ($8,500 / 4.7 days). This implies that roughly 76% of spending attributable to AEs results from the baseline cost of operating an inpatient hospital bed for a longer period of time. Our secondary analysis of costs separated by grouped functional centre showed that hospital spending attributable to AEs was largely driven by nursing and ICU costs (Suppl. Table 5B, Additional File 2), which are both closely related to LOS; however, these costs were also concentrated in unplanned admissions and spine admissions. Given that LOS in these subgroups has not markedly changed since the study period, and that ICU costs are relatively difficult to mitigate or modify, the total costs attributable to AEs in orthopaedic cases may not be greatly affected by current protocols for shorter admissions.

Additionally, our analysis was limited to the in-hospital episode of care; consequently, not all AE-related costs were included in this analysis: for example, costs of post-discharge unplanned physician or emergency department visits, readmission, and planned follow-up clinic visits were not considered; nor were societal costs such as patient income loss and reduced productivity. Furthermore, our costing data did not include physician billings. Therefore, this economic analysis should be considered carefully alongside independent patient risk and system factors, and measures of direct and indirect costs.

Finally, our study took place in Ontario, Canada within a publicly funded single-payer universal healthcare system. The cost and LOS estimates from this cohort may not be generalizable to other jurisdictions or funding models.

## Conclusion

This is the first study to report on a comprehensive cost analysis of AEs in orthopaedic hip, knee, and spine surgery admissions. Adjusted analysis found that AEs were accountable for significant increases in both hospital cost per admission and length of stay. In our study sample of 3,063 patients, 30% experienced one or more AEs during their admission and these events accounted for 14% of hospital expenditures and 19% of all bed-days within the sample. Unplanned orthopaedic admissions had a disproportionately high AE rate and the incremental cost and bed-days associated with AEs in an unplanned admission were almost five times that of a planned admission, which warrants focused investigation of possible mitigation strategies in perioperative risk optimization for unplanned urgent and emergent orthopedic procedures. Our findings may be applicable to other surgical subspecialities, and require broader validation.

### Electronic supplementary material

Below is the link to the electronic supplementary material.

**Additional File 1:** Supplemental information. Document with additional details on OrthoSAVES system, missing data methodology, sensitivity analysis, and adverse event propensity models.

**Additional File 2:** Secondary analysis of costs separated by grouped functional centre. Supplemental tables of unadjusted and propensity-matched cost estimates, broken down by grouped functional centre.

## Data Availability

The data underlying the findings of this study contain protected health information. Any data shared externally will be fully deidentified, including recoding or removal of potentially identifying elements (e.g. variables with small cell sizes). Readers can request access to the data by completing a data request following a Schroeder Arthritis Institute Research Committee and UHN Ethics Committee approved research proposal and a data access agreement, signed by all parties (contact via Christian Veillette; Christian.Veillette@uhn.ca).

## Abbreviations

ACS NSQIP: American College of Surgeons National Surgical Quality Improvement Program
AE: Adverse event
ASA: American Society of Anesthesiologists
BMI: Body-mass index
FC: Functional centre
ICU: Intensive care unit
LOS: Length of stay
OCCI: Ontario Case Costing Initiative
OECD: Organization for Economic Cooperation and Development
OR: Operating room
OrthoSAVES: Orthopaedic Surgical AdVerse Event Severity
QI: Quality initiative
SAVES: Spine AdVerse Event Severity
