# Supplementary material for: Hospital spending and length of stay attributable to perioperative adverse events for inpatient hip, knee, and spine surgery: a retrospective cohort study
Source: BMC Health Serv Res. 2023 Oct 25;23:1150. doi: 10.1186/s12913-023-10055-z (PMC10598977; doi:10.1186/s12913-023-10055-z)
Supplement: Supplementary file 1 — Additional File 1: Supplemental information. Document with additional details on OrthoSAVES system, missing data methodology, sensitivity analysis, and adverse event propensity models. [file 12913_2023_10055_MOESM1_ESM.docx]

**Additional File**

# Supplemental information for “Hospital spending and length of stay attributable to perioperative adverse events for inpatient hip, knee, and spine surgery: a retrospective cohort study”

[1. OrthoSAVES system 2](#_Toc138246673)

[Supplemental Table 1‑A: Existing literature on SAVES/OrthoSAVES characteristics 2](#_Toc138246674)

[2. Missing data methodology 6](#_Toc138246675)

[Supplemental Table 2‑A: Characteristics of incomplete cases, complete cases, and imputed sample 6](#_Toc138246676)

[3. Sensitivity analysis: Gamma-log regression 14](#_Toc138246677)

[Supplemental Table 3‑A: Gamma-log link regression models for cost and LOS, overall and by site 14](#_Toc138246678)

[Deriving incremental cost and LOS estimates using gamma log-link regression results 22](#_Toc138246679)

[Supplemental Table 3‑B: Incremental cost and LOS attributable to adverse events (AEs), based on gamma-log regression results 23](#_Toc138246680)

[4. Adverse event propensity models 25](#_Toc138246681)

[Supplemental Table 4‑A: Logistic regression models estimating propensity of adverse event, overall and by operative site 25](#_Toc138246682)

[References 27](#_Toc138246683)

###### OrthoSAVES system

Supplemental Table 1‑A: Existing literature on SAVES/OrthoSAVES characteristics

| **Study** | **Tool** | **Study Population** | **Design** | **Outcome(s)** | **Results** |
| --- | --- | --- | --- | --- | --- |
| Chen et al., 2017 (1) Can Surgeons Adequately Capture Adverse Events Using the Spinal Adverse Events Severity System (SAVES) and OrthoSAVES? | SAVES-V2 (spine surgery) and OrthoSAVES (hip, knee, and shoulder surgery) | 164 orthopaedic patients (48 spine, 51 hip, 34 knee, 31 shoulder) having elective surgery at an academic hospital in Canada | Surgeons and non-MD reviewers (with access to clinical notes and hospital personnel other than the surgeon) independently recorded adverse events up to discharge and graded their severity | Inter-rater reliability: surgeons vs. non-MD reviewers | Agreement was adequate for major adverse events (grade 3+), but surgeons underreported minor adverse events compared to non-MD reviewers (p<0.001). |
| Rampersaud et al., 2016 (2) Spinal Adverse Events Severity System, version 2 (SAVES-V2): inter- and intraobserver reliability assessment | SAVES-V2 | 22 cases (10 trauma, 12 degenerative) cases were presented to 51 members of the former Spine Trauma Study Group (STGS) and the Degenerative Spine Study Group (DSSG) in round 1 to assess interoberserver reliability, and 36 members in round 2 to assess intraobserver reliability. | Multicenter, multidisciplinary (neurosurgery and orthopedic surgery) inter and intraobserver reliability assessment using case vignettes was completed at consecutive meetings of the STSG and the DSSG. | Inter- and intra-rater reliability among both degenerative spine and spine trauma surgery cases | Agreement on the presence of AEs ranged from 97% to 100% in the 2 groups, while severity classification showed substantial interobserver (ICC = 0.75 for both groups) and intraobserver agreement (ICC = 0.70 in DSSG, 0.71 in STSG). High interobserver agreement and moderate intraobserver agreement was found in both groups on the number of AEs. |
| Karstensen et al., 2016 (3) Morbidity and mortality of complex spine surgery: a prospective cohort study in 679 patients validating the Spine AdVerse Event Severity (SAVES) system in a European population | SAVES | 679 patients undergoing spinal surgery in a tertiary care centre in Denmark. | Prospective cohort study of patients undergoing spinal surgery at an academic tertiary referral center. Research staff collected data using the SAVES form. | AE rate in complex spine surgery at a Danish hospital | One hundred and sixty two (20% incidence) intraoperative adverse events and 1415 (77% incidence) post operative adverse events were reported. |
| Hartig et al., 2015 (4) Adverse events in surgically treated cervical spondylopathic myelopathy: a prospective validated observational study | SAVES | 104 patients undergoing surgery for  cervical spondylopathic myelopathy (CSM) at an academic quaternary referral centre in Canada | Prospective cohort study of patients udergoing CSM surgery at an academic quaternary referral centre. Data was collected using the SAVES tool. | AE rate in surgical treatment of cervical spondylotic myelopathy | A total AE rate of 42.3% was recorded. This included intraoperative (13.5%) and postoperative (37.5%) AE rates. |
| Dea et al., 2014 (5) Adverse events in emergency oncological spine surgery: a prospective analysis | SAVES-V2 | 101 patients undergoing emergency surgery for metastatic spine disease at a quaternary referral centre in Canada. | Prospective cohort study of patients undergoing emergency surgery for metastatic spinal disease at a quaternary referral centre. Data was collected using the SAVES tool at weekly morbidity and mortality rounds. | AE rate in emergency oncological spine surgery cases | Seventy six (76.2%) had at least one AE and 11 died during admission. Intraoperative surgical AEs occurred in 32% of patients. |
| Glennie et al., 2014 (6) Reliability of the spine adverse events severity system (SAVES) for individuals with traumatic spinal cord injury | Spine Adverse Events Severity System for Spinal Cord Injury (SAVES-SCI) | Ten hypothetic patient cases which were completed by 10 raters (7 physicians, 1 nurse, 1 physiotherapist, and 1 researcher) | Raters were presented ten hypothetical cases and test-retest reliability was assessed using the SAVES tool. | Inter- and intra-rater reliability among traumatic spinal injury surgery cases | Intra-rater reliability was high (kappa > 0.6) for identifying and grading all AEs except bone implant, diathermy burn, massive blood loss, myocardial infarction, neurological deterioration, pressure ulcer, return to OR and requirement for tracheostomy. Interrater reliability was good (ICC > 0.6) for identifying and grading intra, pre, and post operative AEs. |
| Street et al., 2013 (7) Use of the Spine Adverse Events Severity System (SAVES) in patients with traumatic spinal cord injury. A comparison with institutional ICD-10 coding for the identification of acute care adverse events | SAVES-V2 | 212 patients in the ICD-10 cohort and 173 patients in the SAVES cohort with spinal cord injuries from a Quaternary Care Spine Program in Canada | Prospective, observational cohort study comparing two cohorts using different systems to record AEs: ICD-10 and SAVES. | Content validity of SAVES-V2 vs. ICD-10 coding among surgery patients with traumatic spinal cord injury | The SAVES system identified twice as many AEs per person than ICD-10 coding. |
| Street et al., 2012 (8) Morbidity and mortality of major adult spinal surgery. A prospective cohort analysis of 942 consecutive patients | SAVES-V2 | 942 patients undergoing spine surgery at an academic quaternary referral centre | Prospective cohort study at an academic quaternary referral centre where SAVES V2 was used to collect AE data during a 12 month period. Data was collected during weekly mortality and morbidity rounds. | - Inter- and intra-rater reliability - AE rate in spine admissions at a Canadian academic hospital | At least one complication was documented for 87% of patients. The rate of intraoperative surgical complication was 10.5% and postoperative complication was 73.5%. |
| Rampersaud et al., 2010 (9) Spine adverse events severity system: content validation and interobserver reliability assessment | SAVES | 200 randomly selected patients during a 1 year period were assessed by 3 raters (staff surgeon, fellow, and/or resident) | Prospective study examining content validity and interobserver relaibility using the SAVES system. | Content validity versus chart review and inter-rater reliability of SAVES among a single surgeon's spine cases | Higher number of surgical AEs using the SAVES form (n=43 vs. n=30) and similar number of medical AEs (n=31 vs. n=27). Substantial agreement was found for the form for number and type of AE when compared to the chart. |

###### Missing data methodology

The R(10) package *mice*(11) was used to generate 20 imputed datasets. Imputation convergence was assessed visually(12), and was judged acceptable with 15 iterations per imputation. The imputation model contained all analysis variables including case cost and length of stay, as well as several auxiliary variables not considered in analysis: smoking status, an indicator for injury caused by acute trauma, day of the week (weekend/holiday versus weekday), and time of operation (morning, afternoon, evening, or night).

Supplemental Table 2‑A: Characteristics of incomplete cases, complete cases, and imputed sample

|  | |  | **Incomplete cases (N = 310)** | | **Complete cases (N = 2753)** | | **Imputed sample (N = 3063)** | |
| --- | --- | --- | --- | --- | --- | --- | --- | --- |
| **Measure** | | **Category** | **Statistic**  [% (count) or mean ± SD] | **95% CI** | **Statistic**  [% (count) or mean ± SD] | **95% CI** | **Statistic**  [% or mean ± SD] | **95% CI** |
| **Analysis variables** | |  |  |  |  |  |  |  |
|  | Anatomical site | Spine | 31.0% (96) | (25.8, 36.1) | 26.9% (740) | (25.2, 28.5) | 27.3% | (25.7, 28.9) |
|  |  | Hip | 56.5% (175) | (50.9, 62.0) | 35.6% (979) | (33.8, 37.3) | 37.7% | (36.0, 39.4) |
|  |  | Knee | 12.6% (39) | (8.9, 16.3) | 37.6% (1034) | (35.7, 39.4) | 35.0% | (33.3, 36.7) |
|  | Sex | Male | 43.9% (136) | (38.3, 49.4) | 45.0% (1238) | (43.1, 46.8) | 44.9% | (43.1, 46.6) |
|  |  | Female | 56.1% (174) | (50.6, 61.7) | 55.0% (1515) | (53.2, 56.9) | 55.1% | (53.4, 56.9) |
|  | Age (years) | Mean ± SD | 68.2 ± 18.3 | (66.2, 70.3) | 63.6 ± 14.2 | (63.1, 64.1) | 64.1 ± 14.7 | (63.6, 64.6) |
|  | Age ≥ 65 | < 65 years | 35.2% (109) | (29.8, 40.5) | 49.8% (1370) | (47.9, 51.6) | 48.3% | (46.5, 50.1) |
|  |  | ≥ 65 years | 64.8% (201) | (59.5, 70.2) | 50.2% (1383) | (48.4, 52.1) | 51.7% | (49.9, 53.5) |
|  | Body-mass index (BMI, kg/m²) | Mean ± SD | 28.1 ± 6.3 (239 missing) | (26.6, 29.6) | 29.3 ± 6.8 | (29.1, 29.6) | 29.0 ± 6.8 | (28.8, 29.3) |
|  | BMI category | Underweight or normal (< 25 kg/m²) | 31.0% (22) | (20.2, 41.7) | 26.0% (716) | (24.4, 27.6) | 27.8% | (26.2, 29.3) |
|  |  | Overweight (25-29.9 kg/m²) | 35.2% (25) | (24.1, 46.3) | 34.5% (951) | (32.8, 36.3) | 34.5% | (32.8, 36.2) |
|  |  | Obese class I/II (30-39.9 kg/m²) | 29.6% (21) | (19.0, 40.2) | 32.5% (896) | (30.8, 34.3) | 31.3% | (29.7, 32.9) |
|  |  | Obese class III (≥ 40 kg/m²) | 4.2% (3) | (0.0, 8.9) | 6.9% (190) | (6.0, 7.8) | 6.4% | (5.6, 7.3) |
|  |  | Missing | — (239) | — | — | — | — | — |
|  | Diagnosis category | Degenerative disorder | 17.6% (48) | (13.1, 22.1) | 71.8% (1977) | (70.1, 73.5) | 66.6% | (64.9, 68.2) |
|  |  | Fracture | 63.0% (172) | (57.3, 68.7) | 10.6% (293) | (9.5, 11.8) | 15.3% | (14.0, 16.6) |
|  |  | Deformity correction | 1.8% (5) | (0.2, 3.4) | 4.0% (110) | (3.3, 4.7) | 3.8% | (3.1, 4.5) |
|  |  | Other | 17.6% (48) | (13.1, 22.1) | 13.5% (373) | (12.3, 14.8) | 14.3% | (13.1, 15.6) |
|  |  | Missing | — (37) | — | — | — | — | — |
|  | Primary/revision procedure | Primary procedure | 88.1% (273) | (84.5, 91.7) | 89.1% (2454) | (88.0, 90.3) | 89.0% | (87.9, 90.1) |
|  |  | Revision or hardware removal | 11.9% (37) | (8.3, 15.5) | 10.9% (299) | (9.7, 12.0) | 11.0% | (9.9, 12.1) |
|  | Procedure | Hip arthroplasty | 26.5% (82) | (21.5, 31.4) | 30.6% (843) | (28.9, 32.3) | 30.2% | (28.6, 31.8) |
|  |  | Hip reduction/fixation | 28.4% (88) | (23.4, 33.4) | 4.5% (124) | (3.7, 5.3) | 6.9% | (6.0, 7.8) |
|  |  | Other hip procedure | 1.6% (5) | (0.2, 3.0) | 0.4% (12) | (0.2, 0.7) | 0.6% | (0.3, 0.8) |
|  |  | Knee arthroplasty | 6.5% (20) | (3.7, 9.2) | 36.1% (995) | (34.3, 37.9) | 33.1% | (31.5, 34.8) |
|  |  | Knee arthroscopic procedure | 1.0% (3) | (0.0, 2.1) | 0.3% (7) | (0.1, 0.4) | 0.3% | (0.1, 0.5) |
|  |  | Knee ligament/tendon repair | 0.6% (2) | (0.0, 1.5) | 0.1% (3) | (0.0, 0.2) | 0.2% | (0.0, 0.3) |
|  |  | Knee reduction/fixation | 3.9% (12) | (1.7, 6.0) | 0.7% (18) | (0.4, 1.0) | 1.0% | (0.6, 1.3) |
|  |  | Other knee procedure | 0.6% (2) | (0.0, 1.5) | 0.4% (11) | (0.2, 0.6) | 0.4% | (0.2, 0.7) |
|  |  | Spine discectomy or decompression | 6.1% (19) | (3.5, 8.8) | 2.8% (77) | (2.2, 3.4) | 3.1% | (2.5, 3.8) |
|  |  | Spine fusion | 20.3% (63) | (15.8, 24.8) | 20.5% (565) | (19.0, 22.0) | 20.5% | (19.1, 21.9) |
|  |  | Spine intradural procedure | 1.6% (5) | (0.2, 3.0) | 1.0% (27) | (0.6, 1.3) | 1.0% | (0.7, 1.4) |
|  |  | Spine osteotomy procedure | 1.9% (6) | (0.4, 3.5) | 1.7% (46) | (1.2, 2.1) | 1.7% | (1.2, 2.2) |
|  |  | Other spine procedure | 1.0% (3) | (0.0, 2.1) | 0.9% (25) | (0.6, 1.3) | 0.9% | (0.6, 1.3) |
|  | Case type | Planned | 25.2% (78) | (20.3, 30.0) | 85.6% (2357) | (84.3, 86.9) | 79.5% | (78.1, 80.9) |
|  |  | Unplanned | 74.8% (232) | (70.0, 79.7) | 14.4% (396) | (13.1, 15.7) | 20.5% | (19.1, 21.9) |
|  | Operating time: incision to close (minutes) | Mean ± SD | 100.2 ± 69.3 (2 missing) | (92.5, 108.0) | 108.2 ± 88.0 | (104.9, 111.5) | 107.4 ± 86.3 | (104.3, 110.4) |
|  | Operating time category | < 1 hour | 35.7% (110) | (30.4, 41.1) | 23.0% (632) | (21.4, 24.5) | 24.2% | (22.7, 25.8) |
|  |  | 1-1.9 hours | 35.7% (110) | (30.4, 41.1) | 51.4% (1414) | (49.5, 53.2) | 49.8% | (48.0, 51.6) |
|  |  | 2-2.9 hours | 13.0% (40) | (9.2, 16.7) | 10.4% (287) | (9.3, 11.6) | 10.7% | (9.6, 11.8) |
|  |  | 3-3.9 hours | 10.7% (33) | (7.3, 14.2) | 7.4% (203) | (6.4, 8.4) | 7.7% | (6.8, 8.6) |
|  |  | 4-4.9 hours | 2.9% (9) | (1.0, 4.8) | 3.7% (102) | (3.0, 4.4) | 3.6% | (3.0, 4.3) |
|  |  | ≥ 5 hours | 1.9% (6) | (0.4, 3.5) | 4.2% (115) | (3.4, 4.9) | 4.0% | (3.3, 4.6) |
|  |  | Missing | — (2) | — | — | — | — | — |
|  | Anatomic site-specific tertile of operating time | Lower | 41.9% (129) | (36.4, 47.4) | 32.2% (886) | (30.4, 33.9) | 33.2% | (31.5, 34.8) |
|  |  | Middle | 28.9% (89) | (23.8, 34.0) | 32.4% (892) | (30.7, 34.1) | 32.0% | (30.4, 33.7) |
|  |  | Upper | 29.2% (90) | (24.1, 34.3) | 35.4% (975) | (33.6, 37.2) | 34.8% | (33.1, 36.5) |
|  |  | Missing | — (2) | — | — | — | — | — |
|  | Preoperative physical status (ASA grade) | 1 (Healthy) | 2.3% (7) | (0.6, 3.9) | 2.7% (75) | (2.1, 3.3) | 2.7% | (2.1, 3.2) |
|  |  | 2 (Mild systemic disease) | 26.5% (82) | (21.5, 31.4) | 42.8% (1177) | (40.9, 44.6) | 41.1% | (39.4, 42.8) |
|  |  | 3 (Severe systemic disease) | 58.7% (182) | (53.2, 64.2) | 49.6% (1365) | (47.7, 51.4) | 50.5% | (48.7, 52.3) |
|  |  | 4 (Life-threatening systemic disease) | 12.3% (38) | (8.6, 15.9) | 4.9% (134) | (4.1, 5.7) | 5.6% | (4.8, 6.4) |
|  |  | 5 (Moribund, not expected to survive without operation) | 0.3% (1) | (0.0, 1.0) | 0.1% (2) | (0.0, 0.2) | 0.1% | (0.0, 0.2) |
|  | Preoperative ASA category | 1-2 | 28.7% (89) | (23.7, 33.7) | 45.5% (1252) | (43.6, 47.3) | 43.8% | (42.0, 45.5) |
|  |  | ≥ 3 | 71.3% (221) | (66.3, 76.3) | 54.5% (1501) | (52.7, 56.4) | 56.2% | (54.5, 58.0) |
|  | Blood transfusion during admission | No | 72.6% (209) | (67.4, 77.7) | 85.0% (2341) | (83.7, 86.4) | 83.9% | (82.6, 85.2) |
|  |  | Yes | 27.4% (79) | (22.3, 32.6) | 15.0% (412) | (13.6, 16.3) | 16.1% | (14.8, 17.4) |
|  |  | Missing | — (22) | — | — | — | — | — |
|  | Discharge disposition | Transferred to other facility, signed out AMA, deceased, or other | 60.3% (187) | (54.9, 65.8) | 32.2% (886) | (30.4, 33.9) | 35.0% | (33.3, 36.7) |
|  |  | Discharged home, with or without support services | 39.7% (123) | (34.2, 45.1) | 67.8% (1867) | (66.1, 69.6) | 65.0% | (63.3, 66.7) |
|  | Comorbid conditions | Hypertension | 47.7% (148) | (42.2, 53.3) | 50.8% (1399) | (48.9, 52.7) | 50.5% | (48.7, 52.3) |
|  |  | Asthma or COPD | 15.5% (48) | (11.5, 19.5) | 16.3% (450) | (15.0, 17.7) | 16.3% | (15.0, 17.6) |
|  |  | Diabetes | 16.5% (51) | (12.3, 20.6) | 16.5% (453) | (15.1, 17.8) | 16.5% | (15.1, 17.8) |
|  |  | Ulcer or stomach disorder | 25.8% (80) | (20.9, 30.7) | 43.6% (1200) | (41.7, 45.4) | 41.8% | (40.0, 43.5) |
|  |  | Kidney disease | 12.3% (38) | (8.6, 15.9) | 11.6% (320) | (10.4, 12.8) | 11.7% | (10.6, 12.8) |
|  |  | Liver disease | 5.8% (18) | (3.2, 8.4) | 5.2% (144) | (4.4, 6.1) | 5.3% | (4.5, 6.1) |
|  |  | Anaemia or other blood disorder | 5.2% (16) | (2.7, 7.6) | 11.2% (307) | (10.0, 12.3) | 10.5% | (9.5, 11.6) |
|  |  | Cancer | 11.6% (36) | (8.0, 15.2) | 8.5% (234) | (7.5, 9.5) | 8.8% | (7.8, 9.8) |
|  |  | Depression | 8.4% (26) | (5.3, 11.5) | 11.1% (305) | (9.9, 12.3) | 10.8% | (9.7, 11.9) |
|  |  | Osteoarthritis | 37.7% (117) | (32.3, 43.1) | 74.0% (2037) | (72.4, 75.6) | 70.3% | (68.7, 71.9) |
|  |  | Chronic back pain | 20.6% (64) | (16.1, 25.2) | 41.7% (1149) | (39.9, 43.6) | 39.6% | (37.9, 41.3) |
|  |  | Rheumatoid arthritis | 1.9% (6) | (0.4, 3.5) | 3.6% (100) | (2.9, 4.3) | 3.5% | (2.8, 4.1) |
|  |  | Coronary artery disease and/or history of heart attack | 9.4% (29) | (6.1, 12.6) | 7.2% (198) | (6.2, 8.2) | 7.4% | (6.5, 8.3) |
|  |  | History of heart failure | 8.4% (26) | (5.3, 11.5) | 7.2% (199) | (6.3, 8.2) | 7.3% | (6.4, 8.3) |
|  |  | History of stroke | 7.7% (24) | (4.8, 10.7) | 5.1% (140) | (4.3, 5.9) | 5.4% | (4.6, 6.2) |
|  |  | Hypercholesterolemia | 26.5% (82) | (21.5, 31.4) | 29.6% (814) | (27.9, 31.3) | 29.3% | (27.6, 30.9) |
|  | Number of comorbid conditions | Mean ± SD (number of conditions) | 2.6 ± 1.9 | (2.4, 2.8) | 3.4 ± 1.9 | (3.4, 3.5) | 3.3 ± 1.9 | (3.3, 3.4) |
|  | Length of admission | Mean ± SD (days) | 11.9 ± 13.8 | (10.3, 13.4) | 6.7 ± 11.5 | (6.3, 7.2) | 7.3 ± 11.8 | (6.8, 7.7) |
|  | Hospital admission cost (2021 $CAD) | Mean ± SD (CAD) | 21803.9 ± 19259.0 (17 missing) | (19598.7, 24009.1) | 17819.4 ± 36197.4 | (16467.2, 19171.5) | 18205.3 ± 34873.5 | (16970.2, 19440.3) |
|  | Number of adverse events (AEs) | None | 63.9% (198) | (58.5, 69.2) | 70.6% (1944) | (68.9, 72.3) | 69.9% | (68.3, 71.6) |
|  |  | 1 | 21.3% (66) | (16.7, 25.8) | 22.4% (617) | (20.9, 24.0) | 22.3% | (20.8, 23.8) |
|  |  | 2 or more | 14.8% (46) | (10.9, 18.8) | 7.0% (192) | (6.0, 7.9) | 7.8% | (6.8, 8.7) |
|  | Any intraoperative AEs | No | 97.4% (302) | (95.7, 99.2) | 96.8% (2664) | (96.1, 97.4) | 96.8% | (96.2, 97.5) |
|  |  | Yes | 2.6% (8) | (0.8, 4.3) | 3.2% (89) | (2.6, 3.9) | 3.2% | (2.5, 3.8) |
|  | Any postoperative AEs | No | 65.2% (202) | (59.9, 70.5) | 72.8% (2004) | (71.1, 74.5) | 72.0% | (70.4, 73.6) |
|  |  | Yes | 34.8% (108) | (29.5, 40.1) | 27.2% (749) | (25.5, 28.9) | 28.0% | (26.4, 29.6) |
|  | Severity grade of worst AE | 0 | 67.1% (198) | (61.8, 72.5) | 70.6% (1944) | (68.9, 72.3) | 69.9% | (68.3, 71.6) |
|  |  | 1 | 1.4% (4) | (0.0, 2.7) | 0.5% (15) | (0.3, 0.8) | 0.6% | (0.3, 0.9) |
|  |  | 2 | 28.1% (83) | (23.0, 33.3) | 23.3% (641) | (21.7, 24.9) | 24.1% | (22.6, 25.6) |
|  |  | 3 | 2.4% (7) | (0.6, 4.1) | 4.8% (131) | (4.0, 5.6) | 4.5% | (3.8, 5.3) |
|  |  | 4 | 0.0% (0) | (0.0, 0.0) | 0.4% (11) | (0.2, 0.6) | 0.4% | (0.1, 0.6) |
|  |  | 5 | 0.3% (1) | (0.0, 1.0) | 0.2% (5) | (0.0, 0.3) | 0.2% | (0.0, 0.4) |
|  |  | 6 | 0.7% (2) | (0.0, 1.6) | 0.2% (6) | (0.0, 0.4) | 0.3% | (0.1, 0.5) |
|  |  | Missing | — (15) | — | — | — | — | — |
|  | Any low-severity AEs (grade < 3) | No | 68.8% (203) | (63.5, 74.1) | 73.8% (2033) | (72.2, 75.5) | 73.0% | (71.4, 74.6) |
|  |  | Yes | 31.2% (92) | (25.9, 36.5) | 26.2% (720) | (24.5, 27.8) | 27.0% | (25.4, 28.6) |
|  |  | Missing | — (15) | — | — | — | — | — |
|  | Any high-severity AEs (grade ≥ 3) | No | 96.6% (285) | (94.5, 98.7) | 94.4% (2600) | (93.6, 95.3) | 94.7% | (93.9, 95.4) |
|  |  | Yes | 3.4% (10) | (1.3, 5.5) | 5.6% (153) | (4.7, 6.4) | 5.3% | (4.6, 6.1) |
|  |  | Missing | — (15) | — | — | — | — | — |
| **Auxiliary variables** | |  |  |  |  |  |  |  |
|  | Smoking status | Current smoker | 49.2% (60) | (40.3, 58.1) | 28.1% (377) | (25.6, 30.5) | 28.6% | (27.0, 30.2) |
|  |  | Quit in the past 12 months | 0.0% (0) | (0.0, 0.0) | 3.6% (49) | (2.6, 4.6) | 3.8% | (3.1, 4.5) |
|  |  | Quit more than 12 months ago | 40.2% (49) | (31.5, 48.9) | 65.6% (881) | (63.0, 68.1) | 62.8% | (61.0, 64.5) |
|  |  | Never smoked | 10.7% (13) | (5.2, 16.1) | 2.8% (37) | (1.9, 3.6) | 4.8% | (4.1, 5.6) |
|  |  | Missing | — (188) | — | — (1409) | — | — | — |
|  | Injury caused by acute trauma | No | 46.3% (143) | (40.7, 51.8) | 90.2% (2482) | (89.0, 91.3) | 85.7% | (84.5, 87.0) |
|  |  | Yes | 53.7% (166) | (48.2, 59.3) | 9.8% (271) | (8.7, 11.0) | 14.3% | (13.0, 15.5) |
|  |  | Missing | — (1) | — | — | — | — | — |
|  | Day of the week | Weekend or statutory holiday | 2.6% (8) | (0.8, 4.3) | 0.2% (5) | (0.0, 0.3) | 0.4% | (0.2, 0.7) |
|  |  | Working weekday | 97.4% (302) | (95.7, 99.2) | 99.8% (2748) | (99.7, 100.0) | 99.6% | (99.3, 99.8) |
|  | Time of day operation started | Morning (6AM-12PM) | 26.6% (82) | (21.7, 31.6) | 54.0% (1486) | (52.1, 55.8) | 51.2% | (49.5, 53.0) |
|  |  | Afternoon (12PM-5PM) | 36.0% (111) | (30.7, 41.4) | 39.9% (1098) | (38.1, 41.7) | 39.5% | (37.8, 41.2) |
|  |  | Evening (5PM-9PM) | 20.5% (63) | (15.9, 25.0) | 2.9% (81) | (2.3, 3.6) | 4.7% | (4.0, 5.5) |
|  |  | Night (9PM-6AM) | 16.9% (52) | (12.7, 21.1) | 3.2% (88) | (2.5, 3.9) | 4.6% | (3.8, 5.3) |
|  |  | Missing | — (2) | — | — | — | — | — |

###### Sensitivity analysis: Gamma-log regression

Multivariable models were estimated for both cost and length of stay outcomes, including an indicator for any adverse event. The same predictors used to model the AE propensity scores were also included in each model. For each model, the cost and LOS ratios for AEs were used to estimate the cumulative case cost and length of stay attributable to AEs for the entire sample, as well as the average incremental cost and length of stay per case (see details on page 22 and resulting estimates in Supplemental Table 3-B).

Supplemental Table 3‑A: Gamma-log link regression models for cost and LOS, overall and by site

|  |  | |  | **Outcome: case cost (2021 $CAD)** | | | **Outcome: length of stay (days)** | | |
| --- | --- | --- | --- | --- | --- | --- | --- | --- | --- |
|  |  | |  | Cost ratio (95% CI) P | | | LOS ratio (95% CI) P | | |
|  | **Measure** | | **Term** | **All cases** | **Planned cases** | **Unplanned cases** | **All cases** | **Planned cases** | **Unplanned cases** |
| **Group: All anatomical sites** | | | |  |  |  |  |  |  |
|  | | **N** | **—** | **3063** | **2435** | **628** | **3063** | **2435** | **628** |
|  | | Intercept | — | 17844 (15781, 20176) <0.001 | 20009 (17515, 22858) <0.001 | 19949 (15587, 25532) <0.001 | 5.09 (4.51, 5.74) <0.001 | 5.67 (5.01, 6.43) <0.001 | 8.68 (6.56, 11.48) <0.001 |
|  | | Age group (ref: <65 years) | ≥ 65 years | 1.09 (1.02, 1.17) 0.011 | 1.08 (1.00, 1.16) 0.045 | 1.08 (0.91, 1.29) 0.388 | 1.16 (1.08, 1.24) <0.001 | 1.14 (1.06, 1.22) <0.001 | 1.13 (0.92, 1.38) 0.235 |
|  | | Sex (ref: male) | Female | 1.00 (0.93, 1.06) 0.905 | 1.02 (0.95, 1.09) 0.676 | 0.91 (0.78, 1.05) 0.200 | 1.04 (0.98, 1.11) 0.229 | 1.06 (0.99, 1.14) 0.071 | 0.91 (0.77, 1.07) 0.238 |
|  | | Adverse event (ref: zero events) | One or more events | 1.35 (1.26, 1.45) <0.001 | 1.25 (1.15, 1.35) <0.001 | 1.61 (1.38, 1.88) <0.001 | 1.56 (1.46, 1.67) <0.001 | 1.47 (1.37, 1.58) <0.001 | 1.72 (1.45, 2.04) <0.001 |
|  | | Anatomical site (ref: spine) | Hip | 0.46 (0.42, 0.49) <0.001 | 0.43 (0.39, 0.47) <0.001 | 0.55 (0.46, 0.65) <0.001 | 0.61 (0.56, 0.66) <0.001 | 0.53 (0.49, 0.58) <0.001 | 0.87 (0.71, 1.07) 0.194 |
|  |  |  | Knee | 0.43 (0.39, 0.47) <0.001 | 0.43 (0.39, 0.47) <0.001 | 0.39 (0.29, 0.52) <0.001 | 0.58 (0.54, 0.64) <0.001 | 0.56 (0.51, 0.61) <0.001 | 0.55 (0.40, 0.75) <0.001 |
|  | | Case type (ref: planned) | Unplanned | 1.64 (1.50, 1.80) <0.001 | — | — | 2.39 (2.19, 2.62) <0.001 | — | — |
|  | | BMI category (ref: normal or underweight [BMI < 25 kg/m^2^]) | Overweight (25-29.9) | 0.92 (0.84, 1.00) 0.051 | 0.87 (0.79, 0.95) 0.003 | 1.07 (0.89, 1.30) 0.474 | 0.93 (0.85, 1.02) 0.140 | 0.90 (0.82, 0.99) 0.024 | 1.04 (0.83, 1.31) 0.715 |
|  |  |  | Obese class I/II (30-39.9) | 0.92 (0.84, 1.01) 0.081 | 0.87 (0.79, 0.96) 0.007 | 1.10 (0.86, 1.40) 0.453 | 0.96 (0.87, 1.05) 0.393 | 0.93 (0.85, 1.02) 0.105 | 1.11 (0.83, 1.49) 0.464 |
|  |  |  | Obese class III (≥ 40) | 0.98 (0.84, 1.13) 0.751 | 0.92 (0.79, 1.08) 0.306 | 1.21 (0.77, 1.89) 0.406 | 1.05 (0.91, 1.21) 0.522 | 0.99 (0.85, 1.14) 0.855 | 1.34 (0.81, 2.20) 0.254 |
|  | | Procedure type (ref: primary procedure) | Revision/hardware removal | 0.91 (0.82, 1.01) 0.072 | 0.95 (0.85, 1.06) 0.323 | 0.81 (0.61, 1.07) 0.135 | 0.95 (0.86, 1.05) 0.322 | 0.97 (0.87, 1.07) 0.537 | 0.90 (0.66, 1.23) 0.510 |
|  | | Pre-operative ASA (ref: grade 1 or 2) | ASA ≥ 3 | 1.19 (1.10, 1.28) <0.001 | 1.15 (1.06, 1.24) <0.001 | 1.44 (1.18, 1.76) <0.001 | 1.13 (1.05, 1.21) 0.001 | 1.10 (1.02, 1.18) 0.009 | 1.29 (1.04, 1.62) 0.023 |
|  | | Anatomic site-specific tertile of operating time (ref: lowest tertile) | Middle tertile | 1.06 (0.98, 1.15) 0.173 | 1.09 (1.00, 1.19) 0.056 | 1.00 (0.84, 1.20) 0.973 | 0.97 (0.90, 1.05) 0.460 | 1.00 (0.93, 1.09) 0.909 | 0.92 (0.76, 1.12) 0.423 |
|  |  |  | Highest tertile | 1.43 (1.32, 1.55) <0.001 | 1.47 (1.35, 1.60) <0.001 | 1.32 (1.11, 1.58) 0.002 | 1.19 (1.10, 1.28) <0.001 | 1.21 (1.12, 1.31) <0.001 | 1.14 (0.94, 1.40) 0.184 |
|  | | Comorbidities | Number of conditions | 1.01 (0.99, 1.03) 0.231 | 1.00 (0.98, 1.02) 0.873 | 1.04 (1.00, 1.08) 0.084 | 1.02 (1.00, 1.04) 0.020 | 1.02 (1.00, 1.04) 0.091 | 1.03 (0.98, 1.08) 0.206 |
| **Group: Hip** | | | |  |  |  |  |  |  |
|  | | **N** | **—** | **1154** | **792** | **362** | **1154** | **792** | **362** |
|  | | Intercept | — | 8071 (7160, 9097) <0.001 | 9259 (8627, 9937) <0.001 | 11468 (8573, 15341) <0.001 | 3.06 (2.63, 3.55) <0.001 | 3.26 (2.91, 3.67) <0.001 | 8.05 (5.70, 11.37) <0.001 |
|  | | Age group (ref: <65 years) | ≥ 65 years | 1.02 (0.95, 1.10) 0.623 | 1.00 (0.96, 1.04) 0.878 | 1.12 (0.87, 1.45) 0.372 | 1.12 (1.02, 1.23) 0.020 | 1.09 (1.02, 1.16) 0.016 | 1.27 (0.94, 1.70) 0.120 |
|  | | Sex (ref: male) | Female | 1.01 (0.94, 1.08) 0.764 | 1.04 (1.00, 1.09) 0.039 | 0.95 (0.78, 1.15) 0.598 | 1.02 (0.93, 1.11) 0.720 | 1.07 (1.00, 1.14) 0.054 | 0.91 (0.72, 1.14) 0.400 |
|  | | Adverse event (ref: zero events) | One or more events | 1.27 (1.18, 1.36) <0.001 | 1.14 (1.10, 1.20) <0.001 | 1.38 (1.14, 1.67) 0.001 | 1.41 (1.29, 1.54) <0.001 | 1.30 (1.21, 1.39) <0.001 | 1.54 (1.22, 1.93) <0.001 |
|  | | Case type (ref: planned) | Unplanned | 1.91 (1.76, 2.08) <0.001 | — | — | 3.01 (2.71, 3.35) <0.001 | — | — |
|  | | BMI category (ref: normal or underweight [BMI < 25 kg/m2]) | Overweight (25-29.9) | 1.02 (0.93, 1.12) 0.620 | 0.97 (0.93, 1.02) 0.261 | 1.09 (0.84, 1.41) 0.512 | 1.02 (0.90, 1.16) 0.722 | 0.96 (0.89, 1.04) 0.349 | 1.11 (0.80, 1.54) 0.514 |
|  |  |  | Obese class I/II (30-39.9) | 0.96 (0.87, 1.07) 0.492 | 0.96 (0.91, 1.01) 0.152 | 0.95 (0.66, 1.38) 0.801 | 0.98 (0.86, 1.11) 0.714 | 0.94 (0.86, 1.03) 0.169 | 1.02 (0.66, 1.58) 0.930 |
|  |  |  | Obese class III (≥ 40) | 1.23 (1.00, 1.52) 0.052 | 1.04 (0.93, 1.16) 0.456 | 2.46 (1.03, 5.88) 0.043 | 1.27 (0.98, 1.65) 0.067 | 1.00 (0.83, 1.19) 0.960 | 2.72 (1.01, 7.31) 0.047 |
|  | | Procedure type (ref: primary procedure) | Revision/hardware removal | 1.05 (0.94, 1.18) 0.411 | 1.14 (1.07, 1.21) <0.001 | 0.77 (0.51, 1.17) 0.226 | 1.09 (0.94, 1.25) 0.256 | 1.13 (1.02, 1.25) 0.023 | 0.96 (0.59, 1.55) 0.853 |
|  | | Pre-operative ASA (ref: grade 1 or 2) | ASA ≥ 3 | 1.12 (1.04, 1.21) 0.003 | 1.08 (1.03, 1.12) <0.001 | 1.28 (0.99, 1.66) 0.058 | 1.09 (0.99, 1.20) 0.072 | 1.08 (1.01, 1.16) 0.026 | 1.08 (0.80, 1.46) 0.609 |
|  | | Joint-specific operating time tertile (ref: lowest tertile) | Middle tertile | 1.01 (0.93, 1.10) 0.825 | 1.01 (0.96, 1.06) 0.776 | 0.94 (0.74, 1.19) 0.608 | 0.95 (0.86, 1.06) 0.385 | 0.97 (0.89, 1.05) 0.411 | 0.88 (0.67, 1.17) 0.386 |
|  |  |  | Highest tertile | 1.12 (1.03, 1.22) 0.010 | 1.08 (1.02, 1.14) 0.005 | 1.16 (0.92, 1.46) 0.215 | 1.03 (0.93, 1.15) 0.533 | 1.02 (0.93, 1.11) 0.719 | 1.05 (0.80, 1.37) 0.743 |
|  | | Comorbidities | Number of conditions | 1.03 (1.01, 1.05) 0.002 | 1.01 (1.00, 1.02) 0.065 | 1.07 (1.02, 1.13) 0.011 | 1.03 (1.00, 1.05) 0.042 | 1.02 (1.00, 1.04) 0.029 | 1.04 (0.98, 1.11) 0.167 |
| **Group: Knee** | | | |  |  |  |  |  |  |
|  | | **N** | **—** | **1073** | **1020** | **53** | **1073** | **1020** | **53** |
|  | | Intercept | — | 8102 (7439, 8825) <0.001 | 8441 (7797, 9137) <0.001 | 5613 (3346, 9415) <0.001 | 3.18 (2.84, 3.57) <0.001 | 3.27 (2.92, 3.66) <0.001 | 3.15 (1.61, 6.19) 0.001 |
|  | | Age group (ref: <65 years) | ≥ 65 years | 1.00 (0.95, 1.04) 0.888 | 0.98 (0.94, 1.02) 0.404 | 1.28 (0.83, 1.98) 0.260 | 1.07 (1.01, 1.14) 0.025 | 1.06 (1.00, 1.12) 0.055 | 1.44 (0.82, 2.53) 0.202 |
|  | | Sex (ref: male) | Female | 1.03 (0.99, 1.08) 0.174 | 1.04 (0.99, 1.08) 0.092 | 0.94 (0.64, 1.37) 0.731 | 1.07 (1.01, 1.14) 0.024 | 1.07 (1.01, 1.14) 0.016 | 0.93 (0.56, 1.53) 0.757 |
|  | | Adverse event (ref: zero events) | One or more events | 1.17 (1.12, 1.23) <0.001 | 1.17 (1.12, 1.23) <0.001 | 1.14 (0.69, 1.88) 0.589 | 1.34 (1.26, 1.43) <0.001 | 1.34 (1.26, 1.43) <0.001 | 1.53 (0.79, 2.95) 0.198 |
|  | | Case type (ref: planned) | Unplanned | 1.28 (1.15, 1.42) <0.001 | — | — | 1.82 (1.58, 2.09) <0.001 | — | — |
|  | | BMI category (ref: normal or underweight [BMI < 25 kg/m2]) | Overweight (25-29.9) | 1.00 (0.93, 1.07) 0.950 | 0.99 (0.93, 1.06) 0.806 | 0.96 (0.60, 1.54) 0.851 | 0.96 (0.87, 1.05) 0.379 | 0.98 (0.89, 1.07) 0.644 | 0.70 (0.37, 1.30) 0.247 |
|  |  |  | Obese class I/II (30-39.9) | 1.02 (0.96, 1.10) 0.494 | 1.00 (0.94, 1.07) 0.954 | 1.35 (0.75, 2.42) 0.300 | 1.02 (0.93, 1.12) 0.606 | 1.03 (0.94, 1.13) 0.485 | 0.90 (0.40, 2.00) 0.781 |
|  |  |  | Obese class III (≥ 40) | 1.06 (0.97, 1.15) 0.233 | 1.05 (0.97, 1.14) 0.238 | 0.97 (0.26, 3.69) 0.965 | 1.08 (0.96, 1.22) 0.184 | 1.11 (0.99, 1.25) 0.079 | 0.77 (0.13, 4.60) 0.765 |
|  | | Procedure type (ref: primary procedure) | Revision/hardware removal | 1.03 (0.95, 1.11) 0.483 | 1.03 (0.95, 1.11) 0.458 | 1.33 (0.74, 2.37) 0.329 | 1.03 (0.93, 1.14) 0.601 | 1.03 (0.92, 1.14) 0.625 | 1.32 (0.62, 2.80) 0.460 |
|  | | Pre-operative ASA (ref: grade 1 or 2) | ASA ≥ 3 | 1.06 (1.01, 1.11) 0.024 | 1.04 (0.99, 1.08) 0.109 | 1.48 (0.90, 2.42) 0.117 | 1.00 (0.94, 1.07) 0.905 | 0.98 (0.92, 1.05) 0.556 | 1.60 (0.85, 3.00) 0.138 |
|  | | Joint-specific operating time tertile (ref: lowest tertile) | Middle tertile | 1.05 (0.99, 1.10) 0.093 | 1.04 (0.99, 1.10) 0.083 | 1.60 (0.86, 2.99) 0.134 | 0.97 (0.91, 1.05) 0.471 | 0.97 (0.90, 1.03) 0.318 | 1.95 (0.87, 4.39) 0.102 |
|  |  |  | Highest tertile | 1.20 (1.14, 1.26) <0.001 | 1.21 (1.15, 1.27) <0.001 | 1.59 (1.01, 2.49) 0.043 | 1.03 (0.96, 1.10) 0.486 | 1.03 (0.96, 1.10) 0.464 | 1.56 (0.87, 2.80) 0.131 |
|  | | Comorbidities | Number of conditions | 1.02 (1.01, 1.04) <0.001 | 1.02 (1.01, 1.03) 0.003 | 1.09 (0.96, 1.25) 0.170 | 1.04 (1.02, 1.06) <0.001 | 1.03 (1.01, 1.05) <0.001 | 1.11 (0.94, 1.31) 0.225 |
| **Group: Spine** | | | |  |  |  |  |  |  |
|  | | **N** | **—** | **836** | **623** | **213** | **836** | **623** | **213** |
|  | | Intercept | — | 14745 (11700, 18584) <0.001 | 14928 (11503, 19373) <0.001 | 18093 (12645, 25889) <0.001 | 4.57 (3.66, 5.72) <0.001 | 4.55 (3.50, 5.92) <0.001 | 7.62 (5.36, 10.83) <0.001 |
|  | | Age group (ref: <65 years) | ≥ 65 years | 1.25 (1.07, 1.47) 0.006 | 1.27 (1.06, 1.53) 0.011 | 1.00 (0.78, 1.28) 0.991 | 1.25 (1.07, 1.46) 0.005 | 1.34 (1.11, 1.61) 0.002 | 0.92 (0.72, 1.18) 0.511 |
|  | | Sex (ref: male) | Female | 0.90 (0.78, 1.04) 0.169 | 0.93 (0.79, 1.11) 0.435 | 0.80 (0.64, 0.99) 0.044 | 0.97 (0.84, 1.12) 0.667 | 1.01 (0.85, 1.20) 0.921 | 0.83 (0.67, 1.02) 0.081 |
|  | | Adverse event (ref: zero events) | One or more events | 1.53 (1.31, 1.79) <0.001 | 1.32 (1.10, 1.58) 0.003 | 2.27 (1.77, 2.91) <0.001 | 1.83 (1.57, 2.13) <0.001 | 1.70 (1.42, 2.05) <0.001 | 2.28 (1.79, 2.91) <0.001 |
|  | | Case type (ref: planned) | Unplanned | 1.53 (1.28, 1.84) <0.001 | — | — | 1.88 (1.58, 2.24) <0.001 | — | — |
|  | | BMI category (ref: normal or underweight [BMI < 25 kg/m2]) | Overweight (25-29.9) | 0.81 (0.68, 0.97) 0.021 | 0.75 (0.60, 0.92) 0.007 | 1.04 (0.79, 1.36) 0.786 | 0.85 (0.71, 1.01) 0.069 | 0.80 (0.65, 1.00) 0.046 | 1.00 (0.76, 1.32) 0.993 |
|  |  |  | Obese class I/II (30-39.9) | 0.84 (0.69, 1.02) 0.076 | 0.77 (0.61, 0.96) 0.023 | 1.04 (0.77, 1.42) 0.787 | 0.88 (0.73, 1.07) 0.192 | 0.82 (0.65, 1.03) 0.088 | 1.11 (0.81, 1.52) 0.499 |
|  |  |  | Obese class III (≥ 40) | 0.86 (0.60, 1.23) 0.417 | 0.86 (0.56, 1.33) 0.502 | 0.86 (0.53, 1.39) 0.525 | 0.93 (0.66, 1.32) 0.693 | 0.92 (0.59, 1.42) 0.694 | 0.94 (0.59, 1.51) 0.804 |
|  | | Procedure type (ref: primary procedure) | Revision/hardware removal | 0.90 (0.73, 1.10) 0.283 | 0.92 (0.74, 1.14) 0.444 | 0.84 (0.56, 1.28) 0.420 | 0.91 (0.75, 1.10) 0.314 | 0.92 (0.74, 1.15) 0.469 | 0.88 (0.59, 1.32) 0.528 |
|  | | Pre-operative ASA (ref: grade 1 or 2) | ASA ≥ 3 | 1.36 (1.14, 1.62) <0.001 | 1.33 (1.10, 1.60) 0.004 | 1.58 (1.14, 2.20) 0.006 | 1.31 (1.11, 1.54) 0.002 | 1.27 (1.05, 1.54) 0.014 | 1.53 (1.11, 2.10) 0.010 |
|  | | Joint-specific operating time tertile (ref: lowest tertile) | Middle tertile | 1.23 (1.04, 1.47) 0.019 | 1.33 (1.07, 1.64) 0.009 | 1.18 (0.93, 1.50) 0.182 | 1.09 (0.92, 1.29) 0.330 | 1.15 (0.93, 1.42) 0.200 | 1.03 (0.81, 1.31) 0.801 |
|  |  |  | Highest tertile | 2.27 (1.90, 2.73) <0.001 | 2.51 (2.04, 3.10) <0.001 | 1.67 (1.25, 2.23) <0.001 | 1.59 (1.33, 1.89) <0.001 | 1.70 (1.37, 2.09) <0.001 | 1.25 (0.95, 1.66) 0.114 |
|  | | Comorbidities | Number of conditions | 0.98 (0.94, 1.02) 0.387 | 0.99 (0.94, 1.04) 0.582 | 0.99 (0.93, 1.06) 0.828 | 1.00 (0.96, 1.04) 0.949 | 1.00 (0.95, 1.05) 0.911 | 1.02 (0.95, 1.08) 0.618 |

CAD = Canadian dollars; CI = confidence interval; ASA = American Society of Anesthesiologists physical status grade; BMI = body-mass index

Deriving incremental cost and LOS estimates using gamma log-link regression results

To estimate the incremental cost/LOS attributable to AEs in dollars and bed-days respectively, the following assumptions were applied.

For each subgroup of anatomical region and case type (corresponding to each model in Suppl. Table 3-A), let $n$ represent the total number of admissions and $n_{AE}$ the number of admissions with AEs. Let $y_{i}$ represent the outcome (cost or LOS) for the i^th^ admission in the subgroup, $x_{i}$ the AE status of the i^th^ admission ($x_{i}$ = 1 if there was an AE during the admission, and 0 if not), and $z_{i}$ a vector of other covariates. If $\beta_{\text{AE}}$ is the gamma regression coefficient corresponding to AE status, then $\text{exp(}\beta_{\text{AE}})$ is the ratio of expected cost or LOS for admissions with AEs versus admissions without AEs for a given $z$:

$$\text{exp(}\beta_{\text{AE}})=\frac{E(y|x=1,z)}{E(y|x=0,z)}$$

Therefore the expected cost/LOS of a non-AE admission $E(y|x=0,z)$ can be expressed as $\frac{E\left( y | x=1,z \right)}{\text{exp(}\beta_{\text{AE}})}$ (1).

The total expected cost or LOS of the subgroup is the sum of expected cost/LOS for admissions with AEs plus the sum for admissions without AEs:

$$\sum_{i=1}^{n} E(y_{i}|x_{i},z_{i})=\left( n_{\text{AE}}\cdot E(y|x=1,z) \right)+ \left( {(n-n}_{\text{AE}})\cdot E(y|x=0,z) \right)$$

Substituting expression (1) for $E\left( y | x=0,z \right):$

$$\sum_{i=1}^{n} E(y_{i}|x_{i},z_{i})=\left( n_{\text{AE}}\cdot E(y|x=1,z) \right)+ \left( {(n-n}_{\text{AE}})\cdot\frac{E\left( y | x=1,z \right)}{\text{exp(}\beta_{\text{AE}})} \right)$$

Solving for $E\left( y | x=1,z \right)$:

$E\left( y | x=1,z \right)= \frac{\sum_{i=1}^{n} E(y_{i}|x_{i},z_{i})}{n_{\text{AE}}+\frac{{(n-n}_{\text{AE}})}{\text{exp(}\beta_{\text{AE}})}}$ (2)

$E\left( y | x=1,z \right)$ can then be calculated using expression (2) with known $n$, $n_{\text{AE}}$, and $\text{exp(}\beta_{\text{AE}})$, and assuming

$\sum_{i=1}^{n} E(y_{i}|x_{i},z_{i})\approx\sum_{i=1}^{n} y_{i}$ for the subgroup.

The incremental cost or LOS attributable to AEs per admission (assuming constant $z$) is $E\left( y | x=1,z \right)-E\left( y | x=0,z \right)$. Applying expression (1), this can be calculated as$E\left( y | x=1,z \right)-\frac{E\left( y | x=1,z \right)}{\text{exp(}\beta_{\text{AE}})}$.

Supplemental Table 3‑B: Incremental cost and LOS attributable to adverse events (AEs), based on gamma-log regression results

|  |  | **Hospital cost attributable to AEs, 2021 $CAD** | | | | | **Hospital bed-days attributable to AEs** | | | | |
| --- | --- | --- | --- | --- | --- | --- | --- | --- | --- | --- | --- |
|  | **Case type** | **Cost ratio for AEs (95% CI)** | **Incremental cost per AE case, $ thousands (95% CI)^a^** | **Cumulative cost attributable to AEs, $ millions (95% CI)^b^** | **% of subgroup cost attributable to AEs  (95% CI)^c^** | **% of total cohort cost attributable to AEs  (95% CI)^d^** | **Length of stay ratio for AEs (95% CI)** | **Incremental bed-days per AE case  (95% CI)^a^** | **Cumulative bed-days attributable to AEs, thousands (95% CI)^b^** | **% of subgroup bed-days attributable to AEs (95% CI)^c^** | **% of total cohort bed-days attributable to AEs (95% CI)^d^** |
| **All anatomical sites** | |  |  |  |  |  |  |  |  |  |  |
|  | All (N = 3063) | 1.35 (1.26, 1.45) | 5.8 (4.4, 7.2) | 5.3 (4.1, 6.7) | — | 9.6 (7.3, 12.0) | 1.56 (1.46, 1.67) | 3.5 (2.9, 4.1) | 3.2 (2.7, 3.7) | — | 14.4 (12.1, 16.8) |
|  | Planned (N = 2435) | 1.25 (1.15, 1.35) | 3.6 (2.3, 4.9) | 2.5 (1.6, 3.4) | — | 4.4 (2.8, 6.0) | 1.47 (1.37, 1.58) | 2.3 (1.8, 2.7) | 1.6 (1.2, 1.9) | — | 7.0 (5.6, 8.4) |
|  | Unplanned (N = 628) | 1.61 (1.38, 1.88) | 14.2 (9.5, 18.9) | 3.3 (2.2, 4.4) | — | 5.9 (4.0, 7.9) | 1.72 (1.45, 2.04) | 8.1 (5.5, 10.8) | 1.9 (1.3, 2.5) | — | 8.5 (5.7, 11.3) |
| **Hip** | |  |  |  |  |  |  |  |  |  |  |
|  | All (N = 1154) | 1.27 (1.18, 1.36) | 3.6 (2.5, 4.7) | 1.3 (0.9, 1.7) | 7.8 (5.3, 10.2) | 2.3 (1.6, 3.1) | 1.41 (1.29, 1.54) | 2.7 (2.0, 3.4) | 1.0 (0.7, 1.2) | 11.5 (8.4, 14.7) | 4.4 (3.2, 5.6) |
|  | Planned (N = 792) | 1.14 (1.10, 1.20) | 1.5 (1.0, 2.0) | 0.3 (0.2, 0.4) | 3.7 (2.5, 4.9) | 0.6 (0.4, 0.8) | 1.30 (1.21, 1.39) | 1.2 (0.8, 1.5) | 0.2 (0.2, 0.3) | 7.3 (5.2, 9.5) | 1.1 (0.8, 1.4) |
|  | Unplanned (N = 362) | 1.38 (1.14, 1.67) | 7.4 (2.9, 11.9) | 1.1 (0.5, 1.8) | 13.9 (5.5, 22.2) | 2.0 (0.8, 3.3) | 1.54 (1.22, 1.93) | 6.3 (2.9, 9.6) | 1.0 (0.5, 1.5) | 18.6 (8.7, 28.3) | 4.3 (2.0, 6.6) |
| **Knee** | |  |  |  |  |  |  |  |  |  |  |
|  | All (N = 1073) | 1.17 (1.12, 1.23) | 1.8 (1.2, 2.4) | 0.5 (0.3, 0.7) | 4.3 (3.0, 5.7) | 0.9 (0.6, 1.2) | 1.34 (1.26, 1.43) | 1.4 (1.1, 1.8) | 0.4 (0.3, 0.5) | 8.2 (6.3, 10.1) | 1.8 (1.4, 2.2) |
|  | Planned (N = 1020) | 1.17 (1.12, 1.23) | 1.8 (1.3, 2.3) | 0.5 (0.3, 0.6) | 4.4 (3.1, 5.6) | 0.8 (0.6, 1.1) | 1.34 (1.26, 1.43) | 1.4 (1.1, 1.7) | 0.4 (0.3, 0.4) | 8.2 (6.3, 10.1) | 1.6 (1.3, 2.0) |
|  | Unplanned  (N = 53) | 1.14 (0.69, 1.88) | 1.8 (-4.3, 9.3) | 0.0 (-0.1, 0.1) | 3.4 (-8.1, 17.8) | 0.0 (-0.1, 0.2) | 1.53 (0.79, 2.95) | 3.5 (-1.6, 9.8) | 0.0 (-0.0, 0.1) | 11.5 (-5.4, 32.4) | 0.2 (-0.1, 0.6) |
| **Spine** | |  |  |  |  |  |  |  |  |  |  |
|  | All (N = 836) | 1.53 (1.31, 1.79) | 14.7 (9.1, 20.5) | 4.1 (2.5, 5.7) | 14.9 (9.2, 20.8) | 7.3 (4.5, 10.2) | 1.83 (1.57, 2.13) | 6.9 (5.1, 8.7) | 1.9 (1.4, 2.4) | 21.6 (16.0, 27.3) | 8.6 (6.4, 10.9) |
|  | Planned (N = 623) | 1.32 (1.10, 1.58) | 8.4 (2.7, 14.2) | 1.8 (0.6, 3.0) | 9.7 (3.2, 16.5) | 3.2 (1.0, 5.4) | 1.70 (1.42, 2.05) | 5.0 (3.2, 6.8) | 1.1 (0.7, 1.4) | 19.3 (12.4, 26.3) | 4.7 (3.1, 6.5) |
|  | Unplanned (N = 213) | 2.27 (1.77, 2.91) | 38.7 (26.4, 50.9) | 2.6 (1.7, 3.4) | 28.2 (19.2, 37.2) | 4.6 (3.1, 6.0) | 2.28 (1.79, 2.91) | 14.7 (10.2, 19.2) | 1.0 (0.7, 1.3) | 28.5 (19.7, 37.2) | 4.4 (3.0, 5.7) |

^a^ See page 22 for details of how incremental cost/LOS per case were derived
^b^ Cumulative cost/bed-days attributable to AEs = (incremental cost/bed-days per case) × (number of admissions with AEs in the specified subgroup)
^c^ % of subgroup cost/ bed-days attributable to AEs = (cumulative cost/bed-days attributable to AEs) / (total cost/bed-days for the specified subgroup)
^d^ % of total cohort cost/bed-days attributable to AEs = (cumulative cost/LOS attributable to AEs) / (total cost/bed-days for the entire cohort: approximately $55.5 million and 22,200 bed-days)
AE = adverse event, CI = confidence interval

###### Adverse event propensity models

Supplemental Table 4‑A: Logistic regression models estimating propensity of adverse event, overall and by operative site

| **Joint** |  | **All cases** | | **Spine** | | **Hip** | | **Knee** | |
| --- | --- | --- | --- | --- | --- | --- | --- | --- | --- |
| N |  | 3063 | | 836 | | 1154 | | 1073 | |
| AUC (95% CI) |  | 0.604 (0.582, 0.626) | | 0.668 (0.629, 0.708) | | 0.631 (0.595, 0.666) | | 0.574 (0.534, 0.613) | |
| **Measure** | **Term** | **OR (95% CI)** | **P** | **OR (95% CI)** | **P** | **OR (95% CI)** | **P** | **OR (95% CI)** | **P** |
| Intercept | — | 0.29 (0.22, 0.39) | <0.001 | 0.21 (0.12, 0.34) | <0.001 | 0.25 (0.16, 0.40) | <0.001 | 0.35 (0.20, 0.59) | <0.001 |
| Anatomical site (ref: spine) | Hip | 0.87 (0.71, 1.06) | 0.175 | — | — | — | — | — | — |
|  | Knee | 0.76 (0.61, 0.95) | 0.014 | — | — | — | — | — | — |
| Sex (ref: male) | Female | 0.89 (0.76, 1.05) | 0.171 | 0.92 (0.67, 1.25) | 0.585 | 0.91 (0.69, 1.19) | 0.472 | 0.75 (0.57, 1.00) | 0.049 |
| Age group (ref: <65 years) | ≥ 65 years | 1.45 (1.22, 1.72) | <0.001 | 1.64 (1.18, 2.29) | 0.004 | 1.59 (1.18, 2.14) | 0.003 | 1.14 (0.86, 1.53) | 0.359 |
| Pre-operative ASA (ref: grade 1 or 2) | ASA ≥ 3 | 1.21 (1.01, 1.45) | 0.043 | 1.08 (0.74, 1.57) | 0.677 | 1.27 (0.94, 1.72) | 0.114 | 1.16 (0.85, 1.59) | 0.348 |
| BMI category (ref: underweight/normal [< 25]) | Overweight (25-29.9) | 0.82 (0.66, 1.02) | 0.075 | 1.00 (0.67, 1.48) | 0.981 | 0.68 (0.49, 0.95) | 0.025 | 0.93 (0.60, 1.45) | 0.746 |
|  | Obese class I or II (30-39.9) | 1.02 (0.81, 1.28) | 0.892 | 1.70 (1.12, 2.58) | 0.013 | 0.97 (0.66, 1.43) | 0.880 | 0.80 (0.52, 1.23) | 0.312 |
|  | Obese class III (≥ 40) | 0.85 (0.58, 1.24) | 0.398 | 1.12 (0.52, 2.42) | 0.764 | 1.20 (0.54, 2.66) | 0.653 | 0.71 (0.39, 1.27) | 0.242 |
| Comorbidities | Number of conditions | 1.03 (0.98, 1.08) | 0.253 | 1.02 (0.93, 1.12) | 0.657 | 1.03 (0.95, 1.11) | 0.461 | 1.00 (0.92, 1.09) | 0.926 |
| Admission type (ref: planned) | Unplanned | 1.41 (1.14, 1.76) | 0.002 | 1.14 (0.78, 1.67) | 0.505 | 1.75 (1.27, 2.41) | <0.001 | 0.88 (0.45, 1.75) | 0.724 |
| Anatomic site-specific tertile of operating time (ref: lowest) | Middle tertile | 1.09 (0.89, 1.33) | 0.401 | 0.95 (0.64, 1.40) | 0.797 | 1.04 (0.74, 1.45) | 0.826 | 1.38 (0.97, 1.95) | 0.072 |
|  | Highest tertile | 1.42 (1.17, 1.73) | <0.001 | 2.64 (1.82, 3.83) | <0.001 | 1.09 (0.78, 1.53) | 0.595 | 1.16 (0.82, 1.64) | 0.390 |
| Procedure type (ref: primary procedure) | Revision/hardware removal | 1.35 (1.06, 1.73) | 0.015 | 1.60 (1.06, 2.41) | 0.025 | 1.26 (0.81, 1.94) | 0.308 | 1.35 (0.83, 2.19) | 0.231 |

AUC = area under the receiver-operator characteristic (ROC) curve; CI = confidence interval; OR = odds ratio; ASA = American Society of Anesthesiologists physical status grade; BMI = body-mass index

###### References

1. Chen BP, Garland K, Roffey DM, Poitras S, Dervin G, Lapner P, et al. Can Surgeons Adequately Capture Adverse Events Using the Spinal Adverse Events Severity System (SAVES) and OrthoSAVES? Clin Orthop Relat Res. 2017 Jan;475(1):253–60.

2. Rampersaud YR, Anderson PA, Dimar JR, Fisher CG, Spine Trauma Study Group and Degenerative Spine Study Group. Spinal Adverse Events Severity System, version 2 (SAVES-V2): inter- and intraobserver reliability assessment. J Neurosurg Spine. 2016 Aug;25(2):256–63.

3. Karstensen S, Bari T, Gehrchen M, Street J, Dahl B. Morbidity and mortality of complex spine surgery: a prospective cohort study in 679 patients validating the Spine AdVerse Event Severity (SAVES) system in a European population. Spine J. 2016 Feb;16(2):146–53.

4. Hartig D, Batke J, Dea N, Kelly A, Fisher C, Street J. Adverse events in surgically treated cervical spondylopathic myelopathy: a prospective validated observational study. Spine. 2015 Mar 1;40(5):292–8.

5. Dea N, Versteeg A, Fisher C, Kelly A, Hartig D, Boyd M, et al. Adverse events in emergency oncological spine surgery: a prospective analysis. J Neurosurg Spine. 2014 Nov;21(5):698–703.

6. Glennie RA, Noonan VK, Fallah N, Park SE, Thorogood NP, Cheung A, et al. Reliability of the spine adverse events severity system (SAVES) for individuals with traumatic spinal cord injury. Spinal Cord. 2014 Oct;52(10):758–63.

7. Street JT, Thorogood NP, Cheung A, Noonan VK, Chen J, Fisher CG, et al. Use of the Spine Adverse Events Severity System (SAVES) in patients with traumatic spinal cord injury. A comparison with institutional ICD-10 coding for the identification of acute care adverse events. Spinal Cord. 2013 Jun;51(6):472–6.

8. Street JT, Lenehan BJ, DiPaola CP, Boyd MD, Kwon BK, Paquette SJ, et al. Morbidity and mortality of major adult spinal surgery. A prospective cohort analysis of 942 consecutive patients. Spine J. 2012 Jan;12(1):22–34.

9. Rampersaud YR, Neary MA, White K. Spine adverse events severity system: content validation and interobserver reliability assessment. Spine. 2010 Apr 1;35(7):790–5.

10. R Core Team. R: A language and environment for statistical computing [Internet]. Vienna, Austria: R Foundation for Statistical Computing; 2019. Available from: https://www.R-project.org/

11. Buuren S van, Groothuis-Oudshoorn K. mice: Multivariate Imputation by Chained Equations in R. Journal of Statistical Software [Internet]. 2011;45(3). Available from: http://www.jstatsoft.org/v45/i03/

12. Buuren S van. Flexible imputation of missing data [Internet]. Second edition. Boca Raton: CRC Press, Taylor & Francis Group; 2018. 415 p. (Chapman and Hall/CRC interdisciplinary statistics series). Available from: https://stefvanbuuren.name/fimd/
